# Exploring determinant factors influencing muscle quality and sarcopenia in Bilbao’s older adult population through machine learning: A comprehensive analysis approach

**DOI:** 10.1371/journal.pone.0316174

**Published:** 2024-12-31

**Authors:** Naiara Virto, Danielle Marie Dequin, Xabier Río, Amaia Méndez-Zorrilla, Begoña García-Zapirain

**Affiliations:** 1 eVida Research Lab, Faculty of Engineering, University of Deusto, Deusto, Spain; 2 Department of Physical Activity and Sport Sciences, Faculty of Education and Sport, University of Deusto, Deusto, Spain; Bursa Ali Osman Sonmez Oncology Hospital, TÜRKIYE

## Abstract

**Background:**

Sarcopenia and reduced muscle quality index have garnered special attention due to their prevalence among older individuals and the adverse effects they generate. Early detection of these geriatric pathologies holds significant potential, enabling the implementation of interventions that may slow or reverse their progression, thereby improving the individual’s overall health and quality of life. In this context, artificial intelligence opens up new opportunities to identify the key identifying factors of these pathologies, thus facilitating earlier intervention and personalized treatment approaches.

**Objectives:**

investigate anthropomorphic, functional, and socioeconomic factors associated with muscle quality and sarcopenia using machine learning approaches and identify key determinant factors for their potential future integration into clinical practice.

**Methods:**

A total of 1253 older adults (89.5% women) with a mean age of 78.13 ± 5.78 voluntarily participated in this descriptive cross-sectional study, which examines determining factors in sarcopenia and MQI using machine learning techniques. Feature selection was completed using a variety of techniques and feature datasets were constructed according to feature selection. Three machine learning classification algorithms classified sarcopenia and MQI in each dataset, and the performance of classification models was compared.

**Results:**

The predictive models used in this study exhibited AUC scores of 0.7671 for MQI and 0.7649 for sarcopenia, with the most successful algorithms being SVM and MLP. Key factors in predicting both conditions have been shown to be relative power, age, weight, and the 5STS. No single factor is sufficient to predict either condition, and by comprehensively considering all selected features, the study underscores the importance of a holistic approach in understanding and addressing sarcopenia and MQI among older adults.

**Conclusions:**

Exploring the factors that affect sarcopenia and MQI in older adults, this study highlights that relative power, age, weight, and the 5STS are significant determinants. While considering these clinical markers and using a holistic approach, this can provide crucial information for designing personalized and effective interventions to promote healthy aging.

## Introduction

Aging is marked by a progressive loss of physical and physiological capacities, resulting in a decline in functions and heightened vulnerability to death [1]. This deterioration stands as the primary risk factor for major human pathologies [2]. The concurrent increase in life expectancy, coupled with a growing elderly population, raises substantial concerns about public health [3]. Consequently, a rising demand emerges for the development of effective solutions to address age-related pathologies, with frailty and sarcopenia taking center stage among the most prevalent geriatric conditions [4, 5]. The universal decline in both muscle quantity and quality with age further intensifies these concerns, highlighting the acute necessity for targeted interventions and tools to mitigate the consequences of age-related muscular deterioration [6].

Frailty and sarcopenia are related yet distinct conditions associated with aging. While sarcopenia primarily affects the musculoskeletal system, frailty is a more multifactorial syndrome [7]. According to the European Working Group on Sarcopenia in Older People (EWGSOP-2), the first criterion indicating the probable presence of sarcopenia is the characteristic decrease in muscle strength, while the reduction in muscle mass and quality confirms the diagnosis. In cases where inferior physical performance is identified, sarcopenia is categorized as severe [8]. The EWGSOP-2 underscores the significance of assessing not just the quantity of muscle, but also its quality [8]. Muscle quality (MQ) encompasses both microscopic and macroscopic alterations in muscle architecture, as well as the functional output per unit of muscle mass [9].

An essential sign of an older adult’s general health is the overall condition of their muscles [10]. Age-related declines in muscle mass and quality are prevalent for everyone, leading to frailty and sarcopenia, reduced independence, compromised quality of life, and heightened mortality risk [6, 9]. It is imperative to comprehend factors influencing muscle quality, alongside actively maintaining and regulating it, to stave off declines in muscle mass, strength, and regenerative capabilities [11].

One of the possible strategies that maintains optimal health during aging is regular physical activity, as aging, even in healthy individuals, is associated with a progressive decline in muscular, neural and cognitive function, leading to deficits in functionality [12]. These interventions result in an improvement of the features of sarcopenia and muscle quality by optimizing changes in body composition, improving strength and mobility, increasing physical activity levels and improving the cardiorespiratory system, among others [13–15].

The mentioned geriatric pathologies have been given special attention due to their prevalence among older individuals and the adverse events they generate [4, 16]. Additionally, early detection holds significant potential, enabling the implementation of interventions that may slow or reverse progression, reduce health costs, and enhance quality of life [8, 16].

In community and clinical settings, developing specific machine learning (ML) models tailored to predict sarcopenia and MQ based on the characteristics of the studied population is presented as a crucial asset. Machine learning, a subset of artificial intelligence, is a computational technique that enables computers to automatically learn from data to identify patterns and make predictions in order to identify key factors contributing to the risk of health concerns such as MQ and sarcopenia [17]. Machine learning is commonly applied in clinical settings for disease diagnosis and prognosis [18]. Several studies have used ML to discover important factors for predicting sarcopenia and frailty [3, 19, 20]. Enhancing the detection of physiological, environmental, social, and lifestyle factors contributing to frailty and sarcopenia in older individuals will refine prediction models and enhance healthcare system policies and practices [21].

Artificial intelligence opens new opportunities to advance personalized medicine and understand relevant characteristics in pathophysiology, with recent research demonstrating its effectiveness [21, 22]. Utilizing these developments in personalized medicine using ML permits the identification of complex patterns in large datasets, the anticipation of health risks, and aids in the design of intervention strategies. Integrating these techniques into clinical practice, this study aims to investigate anthropomorphic, functional and socioeconomic factors associated with muscle quality and sarcopenia, using ML approaches.

## Methods

### Study design and population

This descriptive cross-sectional study investigates dynamometric, anthropometric, and Short Physical Performance Battery (SPPB) test outcomes, including balance, gait speed test and chair stand test alongside socioeconomic index data. Participants were selected from the "Health for the Elderly" program sponsored by the Bilbao City Council. Inclusion criteria for participants encompass being aged 60 or older, currently enrolled in the "Health for the Elderly" program, and voluntarily participating, with the inability to walk independently serving as the exclusion criteria. Most of the patients are of Basque origin, sharing similar demographic, racial, and body characteristics. This homogeneity in the sample may limit the generalizability of the model, as it does not adequately capture the variability found in a more diverse population. The data collection of the study was approved by the University of Deusto Ethics Committee (reference # ETK-32/18–19), started on May 1st, 2019 and finished on May 31st, 2019 and written informed consent was obtained from each participant prior to study.

### Ethics approval

The data collection of the study was approved by the University of Deusto Ethics Committee (reference # ETK-32/18–19) and written informed consent was obtained from each participant prior to study.

### Measurement protocol

#### Manual grip strength

A Camry EH101 electronic hand dynamometer, approved as medical equipment by the Spanish Agency for Medicines and Health Products, was used to measure hand grip strength. The testing protocol involved individuals adopting a posture with a slight shoulder abduction (approximately 10॰), with the elbow in full extension and the forearm and hand in a neutral position [23]. Each participant underwent two tests, with the higher recorded value used for analysis. The CAMRY EH101 dynamometer, employed in this study, demonstrated excellent reliability and validity. This device stands out as a reliable, cost-effective, and practical tool for evaluating grip strength in geriatric clinical settings [24].

#### Anthropometry

Body composition variables were analyzed using segmental bioimpedance with the Tanita BC-601 segment analyzer. This approach yields information regarding body fat percentage, weight, and muscle mass (kg). The Tanita BC-601 is a reliable non-invasive method that offers precise measurements [25]. Furthermore, the Tanita HR 001 Leicester portable stadiometer was utilized for measuring height.

#### Socioeconomic index

The socioeconomic and physical environment of a region are interconnected with health statistics, exerting a direct impact on the well-being of the elderly population [26]. The Euskadi 2021 socioeconomic index was created using the same approach as the MEDEA project [27]. This variable’s definition is based on the average personal income (in euros) for each type of financial category throughout city neighbourhoods in the municipality of Bilbao in 2021. As a result, three socioeconomic indices, low rent (<20,000 euros), medium rent (>20,000 and <30,000 euros), and high rent (>30,000 euros), have been identified [28].

#### Functional assessment: SPPB

The Short Physical Performance Battery (SPPB) is a commonly used clinical assessment tool renowned for its strong reliability and validity, reporting an excellent inter-rater reliability and test-retest reliability [29, 30]. The SPPB consists of three components: a balance assessment (including standing, semi-tandem, and tandem positions), a four-meter walk test (measuring the time taken to walk four meters at a normal pace), and a five-repetition sit-to-stand test (*5STS*) performed as rapidly as possible [31]. Each component is scored from zero to four, with zero representing the lowest score. Moreover, a combined score, which ranges from zero to 12 points, is calculated by adding together the scores obtained from the three components [31].

#### Relative power

The 5STS test, utilized to assess lower extremity muscle power in clinical or field environments, demonstrates good intra-rater, inter-rater, and test-retest reliability, making it a dependable measurement tool suitable for both experienced and inexperienced raters [32]. To compute the mean absolute value, the equation developed by Alcazar et al., 2018 was utilized, taking into account performance in the 5STS (measured as time to complete five STS repetitions), body mass, body height, and chair height [33]. To standardize the data, the result was divided by body weight [33].

#### Muscle Quality Index (MQI)

In this research, muscle quality was indirectly evaluated using the method proposed by Barbat-Artigas et al. and Chang et al. [34, 35], which involves dividing handgrip strength by relative skeletal muscle mass. The classification of muscle quality was based on the thresholds established by Barbat-Artigas et al. (2012). Participants are categorized into normal (>1.53), low (>1.36 and ≤1.53) and poor (≤1.36) muscle quality for men, and normal (>1.53), low (>1.35 and ≤1.53) and poor (≤1.35) muscle quality for women [35].

Within the data this feature is referred to as *cut off points* with integer values one through three; one corresponding to normal muscle quality and three corresponding to poor muscle quality. This feature is subsequently used for feature selection and classification tests of MQI.

#### Sarcopenia

For sarcopenia tests the feature *sarcEWGSOP* was used as the dependent variable. This feature represents the level of sarcopenia with integer values zero through two: zero corresponding to the absence of sarcopenia, one with moderate presence, and two with severe sarcopenia, as defined by EWGSOP-2 [8]. The calculation of sarcopenia levels was carried out using the gender-specific cut-off values for males and females, according to the EWGSOP-2 criteria.

#### Frailty

Frailty in older adults can be operationally defined using the Short Physical Performance Battery (SPPB), which provides a total score ranging from 0 to 12 points. This score enables the classification of frailty into four categories: non-frail (10–12 points), pre-frail (7–9 points), mild to moderate frailty (4–6 points), and severe frailty (0–3 points) [31].

#### Data preparation and preprocessing

We prepared the data for analysis, including separate processes for the MQI and sarcopenia tests. The raw dataset contained 1,253 individual entries with 39 features. The key preprocessing steps involved data cleaning, filtering relevant variables, applying one-hot encoding to nominal variables, and scaling ordinal variables to ensure comparability. A normalised and standardised dataset was then created to facilitate feature selection using support vector machines.

*Specific processing for MQI*. Seventeen variables were selected for MQI tests: *socioeconomic index (3)*, *age*, *weight*, *bmi*, *fat mass*, *balance 1*, *balance 2*, *balance 3*, *gait speed (m/s)*, *4m test*, *5STS*, *mean power*, *relative power*, *SPPB*, *frailty*, *groups*, *sarcopenia (*v). One-hot encoding was applied to nominal variable *sarcopenia (v)*. Ordinal features *socioeconomic index (3)*, *frailty*, and *groups* were scaled.

*Specific processing for sarcopenia*. Eleven variables were selected for sarcopenia tests: *socioeconomic index (3)*, *sex*, *age*, *height*, *weight*, *bmi*, *fat mass*, *muscle*, *5STS*, *relative power*, *points balance*. *Socioeconomic index (3)* was scaled.

#### Feature selection methods

Feature selection in ML is the process of selecting features relevant for training a prediction model. Feature selection provides many benefits, including reducing computation time, improving prediction performance, and a better understanding of the data in machine learning [36, 37]. A common problem within medical ML studies is small sample size [38]. Feature selection techniques have been shown to provide possible solutions to this, while also helping medical researchers identify the underlying mechanisms that relate to diseases [39, 40].

Four methods were employed in this study for feature selection: Spearman correlation, Ordinary Least Squares (OLS), Random Forest (RF), and Support Vector Machine (SVM). A subset of the data was created using the chosen features from each method. These subsets are subsequently used for classification tests.

*Spearman correlation*. Spearman correlation was used as a filter method to select a subset of the features based on the relationship between the features and the target class. We chose to use Spearman correlation because it is suited to handle data such as the one used in this study which contains both non-normally distributed continuous data and ordinal data [41]. The strength of the relationship between the features and target class was measured using the correlation coefficient. Previous works were utilized for interpreting the correlation coefficient [41, 42].

The following steps were followed to select features using Spearman correlation:

1. Compute the correlation between all variables with the target variable.
2. Select those with an absolute value of the correlation coefficient above a threshold of 0.3. Anything less was considered a weak correlation.
3. Check selected variables for correlation with each other. From each pair of features that are strongly correlated, remove the one that is less correlated with the target. Anything with a coefficient above 0.5 is considered strongly correlated.

*OLS*. An OLS wrapper method was used by iteratively selecting a subset of features and passing it to the model. Based on feature performance another set of features is selected or the process terminates [43]. Feature performance was evaluated based on p-value. The feature with the highest p-value was removed from the selected features. This process was repeated until there were no more features with a p-value above 0.5.

*Random forest*. An embedded method with a random forest ML model was employed to extract features based on importance. Embedded methods use intrinsic properties of the classifier to select the subset of features [43]. We used the meta-transformer *SelectFromModel* from *Scikit-Learn* to select features based on importance weights. Feature importances were computed as the mean and standard deviation of accumulation of the impurity decrease within each tree in the random forest [44].

*SVM*. Feature selection using SVM recursive feature elimination was performed using a linear kernel, inspired by previous work [45]. Features were ranked using the sum of the absolute value of the coefficients. In this process, the feature with the lowest rank is removed and a model is trained on the remaining features. When evaluating the weights of the features we found that the lowest ranked 35% of features had a significantly lower weight. Therefore, we decided to repeat the process until less than 65% of the features remain.

#### Aggregated select features and importance rank

The selected features were ranked and aggregated to create a list of features. The purpose was to investigate model performance with a different number of features from this aggregated list of selected features.

Feature rank is calculated as follows:

1. Each feature is given a rank per selection method. If it is not chosen for a method, it is given the rank of “last place”. For example, in the MQI dataset there are 17 features, and any unchosen feature is given a rank of 17.
2. The *rank sum* for each feature is the sum of all rankings across all methods.
3. The aggregated feature rank is calculated as *one* divided by *rank sum*.

Subsets of data were then created based on the first four, first eight, and the full list of the features from the select feature list. These subsets were then compared in classification tests, alongside the subsets created from each individual selection method, to investigate if using more features provides a better performing model.

#### Sarcopenia and MQI classification modeling using machine learning

Using the subsets of data created from feature selection techniques mentioned above we aimed to see if a particular method for selecting features was ideal to establish a prediction model for both sarcopenia and MQI. For the tests, we used eight ML algorithms including K-nearest Neighbors (KNN), Gradient Boosting (GB), Decision Tree (DT), Gaussian Naive Bayes (NB), Stochastic Gradient Descent (SGD), Random Forest (RF), Multi-Layer Perceptron (MLP), and Support Vector Machine (SVM). All of the models employed come from the Sci-Kit Learn library. Generally, deep learning (DL), a subfield of ML, offers multiple benefits over traditional ML methods [46]. However, when training on small datasets, such as in our case, ML models are preferred over DL models, since a dataset with less than 100,000 samples is considered insufficient for DL [47].

#### Classification experiments

Two steps were employed to compare classification results using the different feature selection methods: baseline test and hyperparameter tuning. All tests were completed using a total of nine datasets. This includes the four subsets of data created to compare classification performance across feature selection methods, as well as the three subsets using the aggregated select features. Classification results are compared against the full dataset as well as a normalized version of the dataset as a baseline. In all tests we chose to perform cross validation instead of a train-test split. Previous works show that, with small datasets such as ours, a train-test split can lead to unreliable test metrics, and therefore cross-validation as a better option [48, 49].

*Baseline test*. For the baseline performance tests we trained a total of 16 models using the aforementioned ML algorithms: eight models for prediction of sarcopenia and eight for MQI. Classifier performance was reported using accuracy, area under ROC curve (AUC) and f1-score. Each model was evaluated based on prediction AUC using test samples from the data. Accuracy is included because it is a commonly reported metric in ML. Our analysis focuses on AUC to compare models due to its ability to handle multiclass classification with class imbalances [50]. F1-score, the harmonic mean between precision and recall, is also reported as it is a common metric in multiclass classification tasks [51]. In both cases we used macro averaging, the arithmetic mean of the score of each class, to not give extra weight to larger classes. For example, a high Macro-F1 value indicates that the algorithm has good performance across all classes [51]. To determine the performance of a given model with each subset of the data created using feature selection, we conducted a five-fold cross-validation, resulting in the average performance across all folds.

Standard parameters were chosen for the baseline experiments, with non-standard parameters used in only a few cases. For all models that have the parameter *random_state* we gave a value of *42* to improve result reproducibility. To ensure reproducibility of models that do not have this parameter, we also set a seed in the environment to *42*. For MLP it was necessary to set the value of *max_iter* to *5000*, as the standard input *200* was not sufficient for convergence. For SVM we used a linear kernel for the baseline experiments, to see if the data are linearly separable before moving on to more complex kernels. To allow for the ability to compute AUC for certain classifiers, we set probability to True for SVM, and used the log_loss function for SGD.

Multilayer perceptrons and SVMs are both sensitive to feature scales [52]. For MLPs standardization is used to increase model performance and decrease the number of epochs required for model convergence [53]. Therefore, all tests involving MLP and SVM include a pipeline with a step to standardize the data before they are fed to the model. The pipeline utilized *StandardScaler* from *Sci-Kit Learn*.

*Hyperparameter tuning and final results*. Default hyperparameters are not guaranteed to give optimal performance [54]. Therefore, using the results of the baseline experiments we selected the three best performing models for further tests to discover the optimal hyperparameters. The three models that we found to have the highest AUC scores for both sarcopenia and muscle quality prediction tasks include RF, SVM, and MLP. With these models we ran hyperparameter tuning using *GridSearchCV* from *Scikit-Learn*, with a five-fold cross-validation. The hyperparameter options tested are shown in Table 1 in S1 File. The best performing models from this stage gave us the final classification results.

**Table 1:** Anthropometric and functional measurements of the study participants.

Variable	Mean ± SD	Med [Min-Max]
Age	78.13 ± 5.78	79 [60–93]
Handgrip Strength (right)	20.7 ± 6.57	20 [6–55]
Muscle Mass (kg)	39.37 ± 5.58	38 [24–65]
Fat Mass (kg)	38.02 ± 5.98	39 [15–64]
BMI	28.44 ± 4.08	28 [16–46]
Gait Speed (m/s)	0.99 ± 0.25	0.99 [0.33–2.94]
Mean Power (*weight*×*BMI*)/5*STS*	121.71 ± 44.5	116.7 [29.24–352.13]
Relative Power (*mean power/kg*)	1.8 ± 0.53	1.74 [0.5–4.46]
SPPB	9.75 ± 1.68	10 [4–12]
MQI	1.25 ± 0.37	1.2 [0.33–3.46]

## Results

### Participant characteristics

A total of 1253 patients aged 60 or older were included in this study: 1121 being female and 132 male. Table 1 shows the anthropometric and functional measurements of the study participants. The degree of disease present within the study population, including MQI, frailty, and sarcopenia is shown in Table 2. Most patients (837) had a poor level of muscle quality, with 201 showing moderate degradation and 215 presenting with healthy MQI. Regarding sarcopenia 513 patients presented as healthy, with 587 showing moderate and 153 showing severe sarcopenia. 748 patients were found to have normal levels of frailty, with 451 showing moderate and 54 showing more advanced levels of frailty.

**Table 2:** Prevalence of disease within the study population.

Variable	Healthy	Moderate	Severe
Sarcopenia (*sarcEWGSOP*)	513	587	153
MQI (*cut off points*)	215	201	837
Frailty	748	451	54

### Feature selection

#### Sarcopenia feature selection frequency and ranking

**Fig 1:**
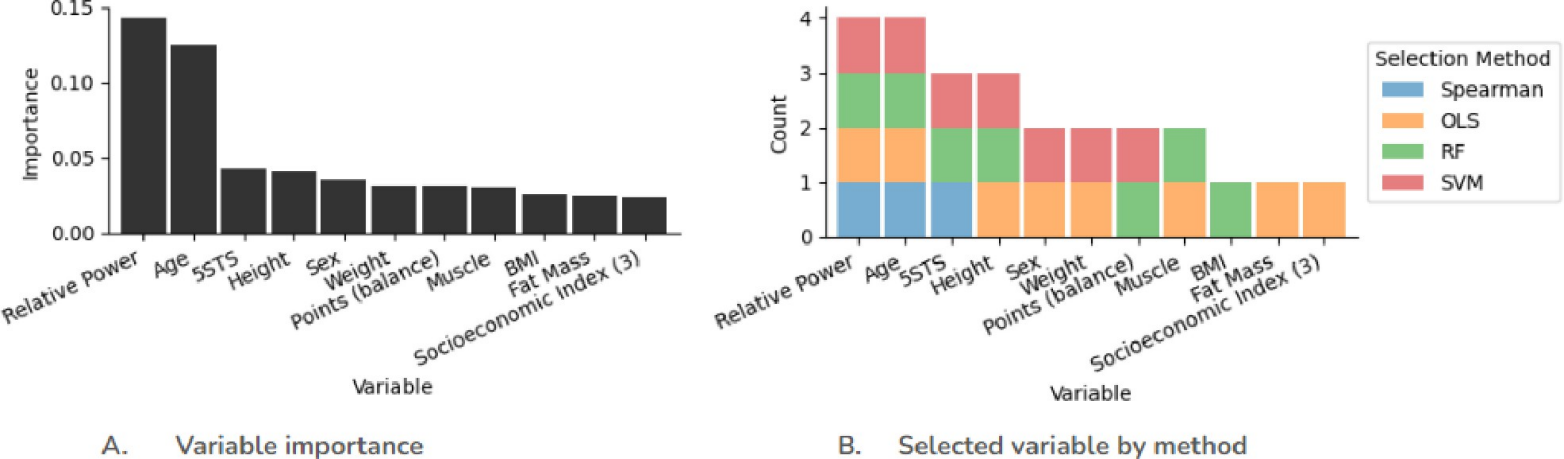
Calculated importance of selected features for sarcopenia.

The calculated importance of the selected features for sarcopenia are shown in Fig 1A. *Relative power* and *age* have a relatively close level of importance for sarcopenia, with an over 50% drop in importance for the next highest ranking feature, *5STS*. The aggregated select features includes all of the features from the full sarcopenia dataset, thereby not reducing the number of features.

#### MQI feature selection frequency and ranking

**Fig 2:**
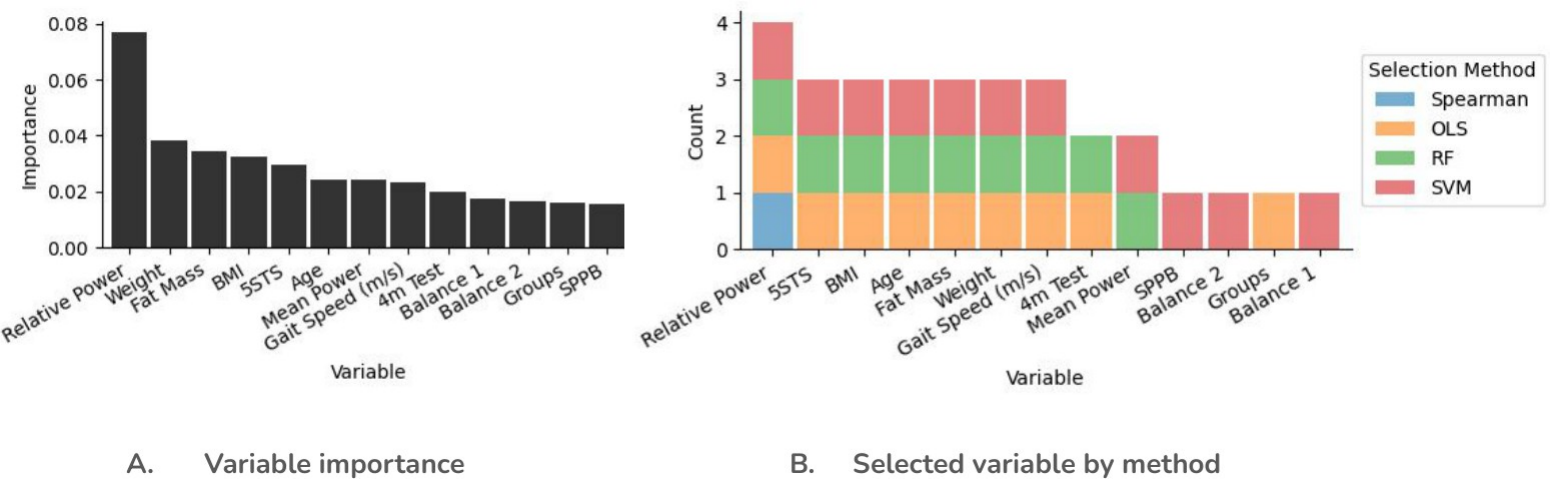
Calculated importance of the features selected based on relevance to MQI.

The calculated importance of each of the features within the aggregated select features for MQI is shown in Fig 2A. The variables in the x-axis are displayed in order of importance, with *relative power* being the most important feature. After *relative power*, the importance of each feature drops by over 50%.

### Classification results

The results of the models are compared using the AUC score for both MQI and sarcopenia classification. Each of the eight ML models was trained on nine datasets separately. Through this we can compare the various selection methods for each disease classification task. A detailed list of the features included in each dataset are shown in Table 4 for MQI and Table 5 for sarcopenia in S1 File.

#### Baseline results

**Fig 3:**
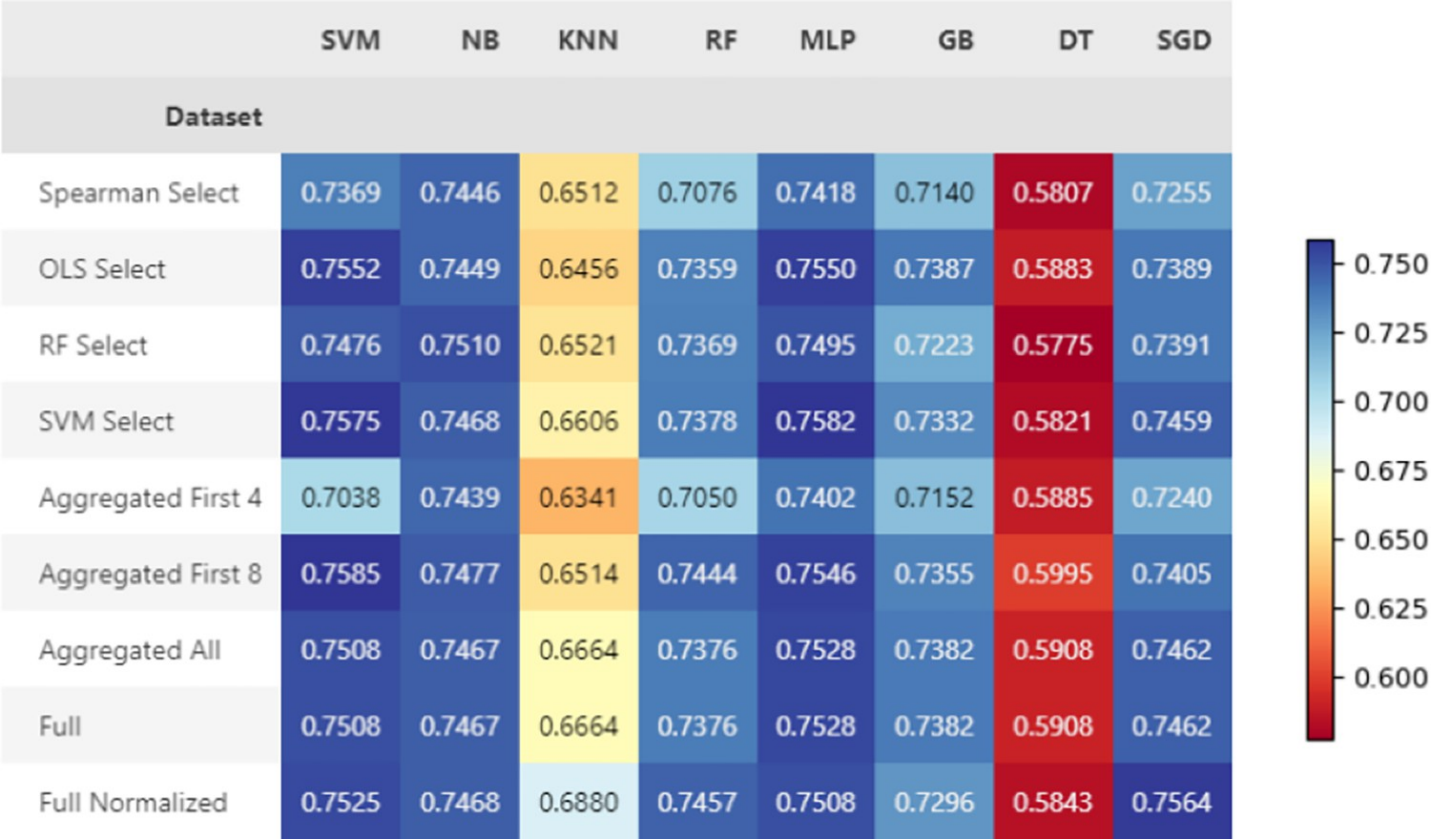
Baseline results for sarcopenia classification, shown in AUC.

**Fig 4:**
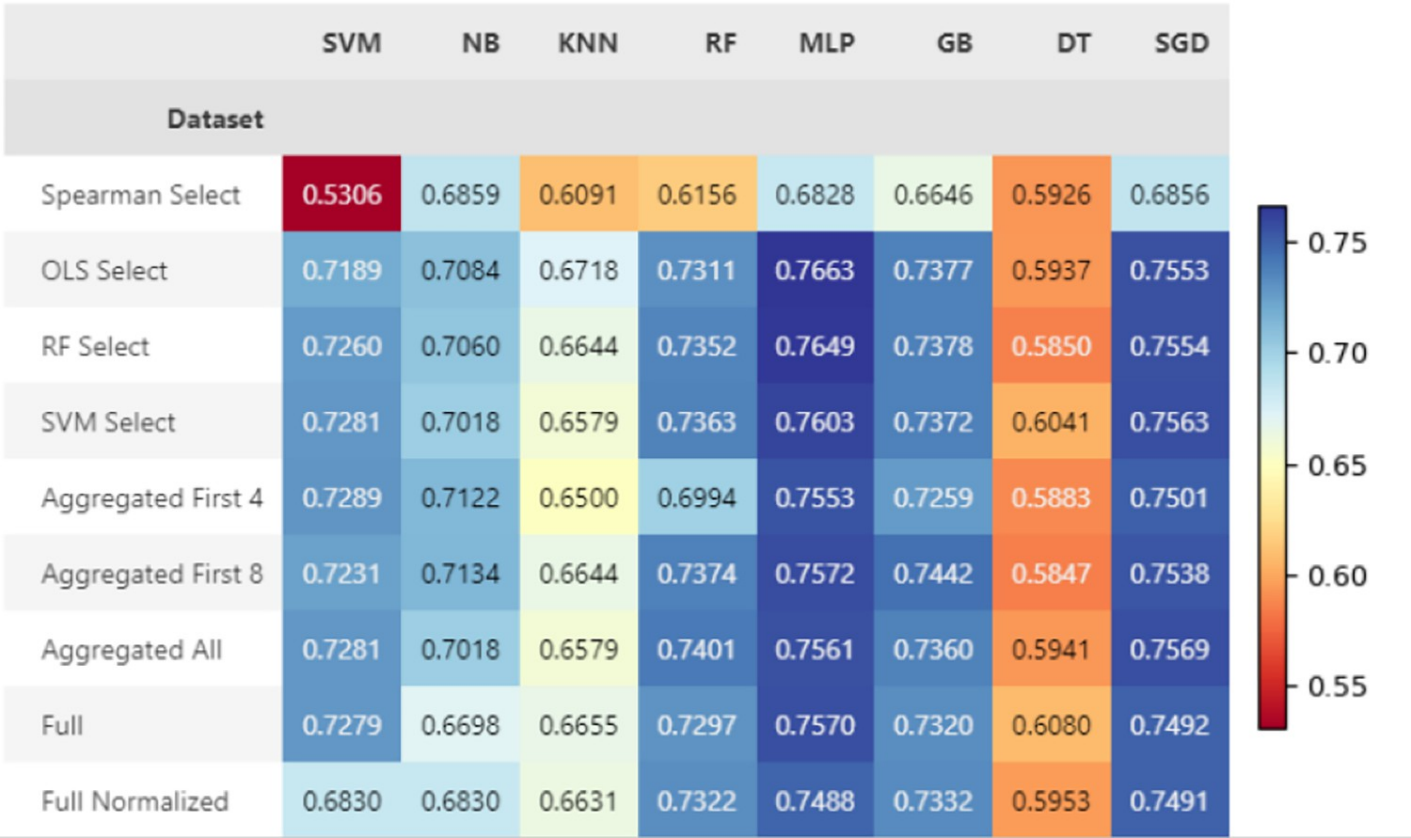
Baseline results for MQI prediction, shown in AUC.

The three models that perform best for both tasks across all datasets are MLP, RF, and SVM. The NB models had similar results to the other highest performing models when classifying sarcopenia, but quite poorer performance when classifying MQI, as shown in Figs 3 and 4. The relatively high AUC values indicate that the models are good at distinguishing between classes by assigning a higher probability to the correct class. Accuracy results are overall higher for MQI prediction, indicating that the models’ ranking ability align closer to their overall ability to classify correctly. The complete performance metrics reported for each test for the baseline experiments are shown Table 6 for sarcopenia and Table 7 for MQI in S1 File.

#### Hyperparameter tuning results

**Fig 5:**
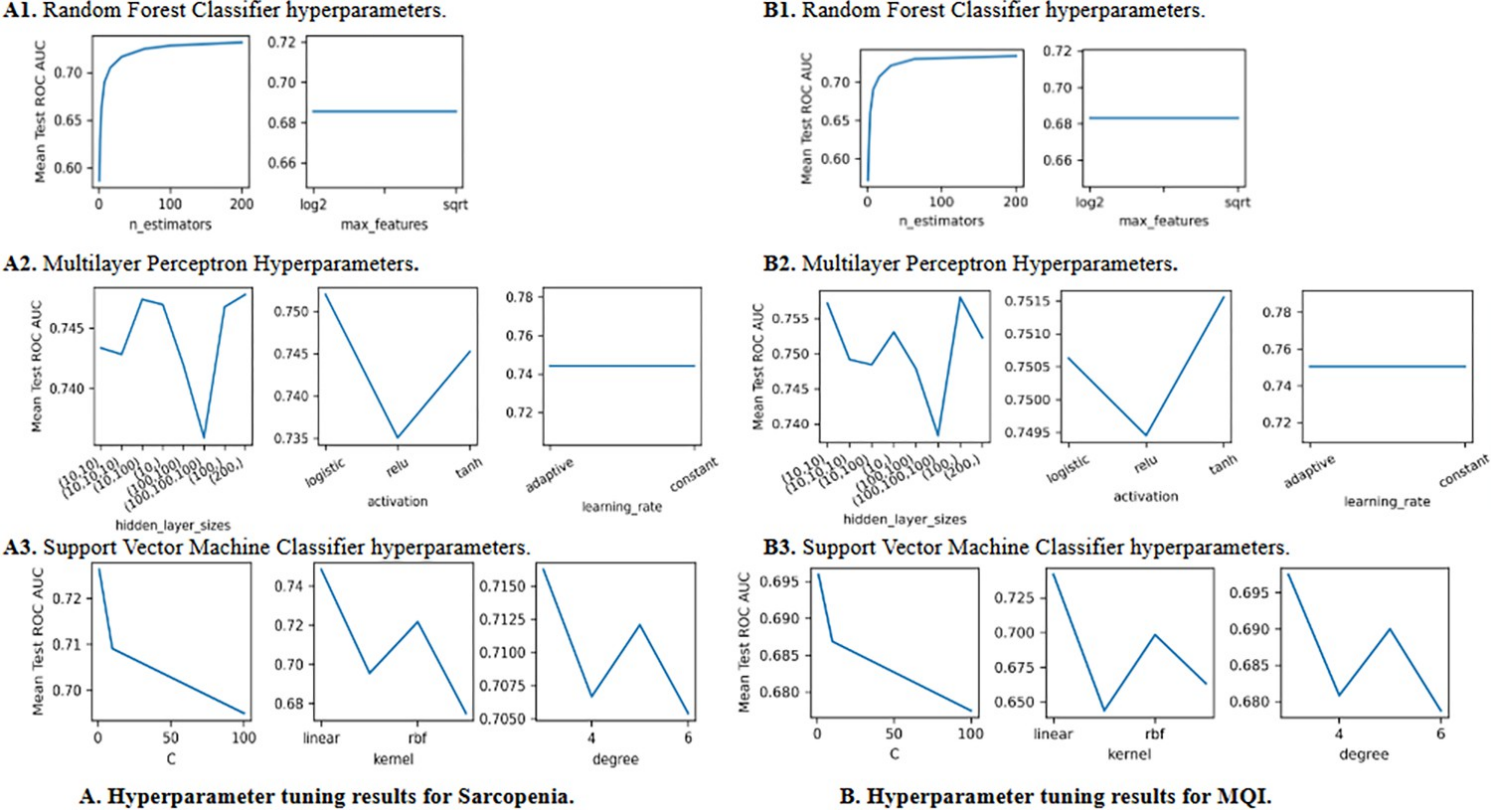
Hyperparameter tuning results for sarcopenia and MQI.

The hyperparameters investigated are as follows: n_estimators and max_features for RF, hidden_layer_sizes, activation, and learning_rate for MLP, and C, kernel, and degree for SVM. The best hyperparameters found for each model and dataset combination are shown in Table 2 for sarcopenia and Table 3 for MQI in S1 File. The results of hyperparameter tuning are shown in Fig A. for sarcopenia and Fig 5B. for MQI. The y-axis in all subplots shows the mean test AUC across all folds using only one of the datasets, with the same process being repeated for each of the nine datasets.

*RF hyperparameters*. A random forest fits a number of DT classifiers on various sub-samples of the dataset and uses averaging to improve the predictive AUC score. The parameter *n_estimators* controls the number of trees in the forest. Increasing the number of trees improves AUC, but also the computation time. It can be seen in both Fig 5A.1. and 5B.1. that model performance increases as this value increases.

*Max_features* is the number of features to consider when looking for the best split. Empirically good values often depend on the type of task. For both MQI and sarcopenia classification, ‘sqrt’ is the best value across the majority of the datasets.

*MLP hyperparameters*. The number of neurons in the neural network are represented by *hidden_layer_sizes*, with the ith value in the tuple representing the number of neurons in the ith layer. For either classification task a larger network does equate to better results, with the best framework for sarcopenia being (100,) or (200,) depending on the dataset, and the best for MQI being (10,10,10) or (200,).

The *activation* parameter represents the activation function for the hidden layer. For sarcopenia a logistic function is shown to be the best activation function, whereas tanh is better for MQI classification.

The *learning_rate* represents the schedule for weight updates. A higher value leads to faster learning by determining the step size taken into the gradient direction in backpropagation. Too small learning rate can lead to very slow learning and increased computation time, while too large a value can lead to early convergence with poor performance. The average test score shown in Fig 5A.2. and 5B.2. do not show a difference between adaptive or constant with the dataset displayed. However, as shown in Table 2 and Table 3 in S1 File a constant learning rate is best.

*SVM hyperparameters*. C is the regularization parameter. It controls the tradeoff of generalizability and overfitting by modifying the width of the margin of the hyperplane between classes. A smaller number corresponds to a larger margin, which will generalize better to unseen data. In the case of sarcopenia and MQI classification the results show a smaller value for *C* being ideal.

The parameter *kernel* specifies the kernel type to be used in the algorithm. The results show a linear algorithm to be the best for both sarcopenia and MQI classification.

When using a polynomial kernel, *degree* specifies the degree of the function. There is not a significant difference in performance using different polynomial degrees, as shown in Fig 5A.3. and 5B.3, however, the best value across all datasets is a degree of three.

#### Final sarcopenia classification results

**Fig 6:**
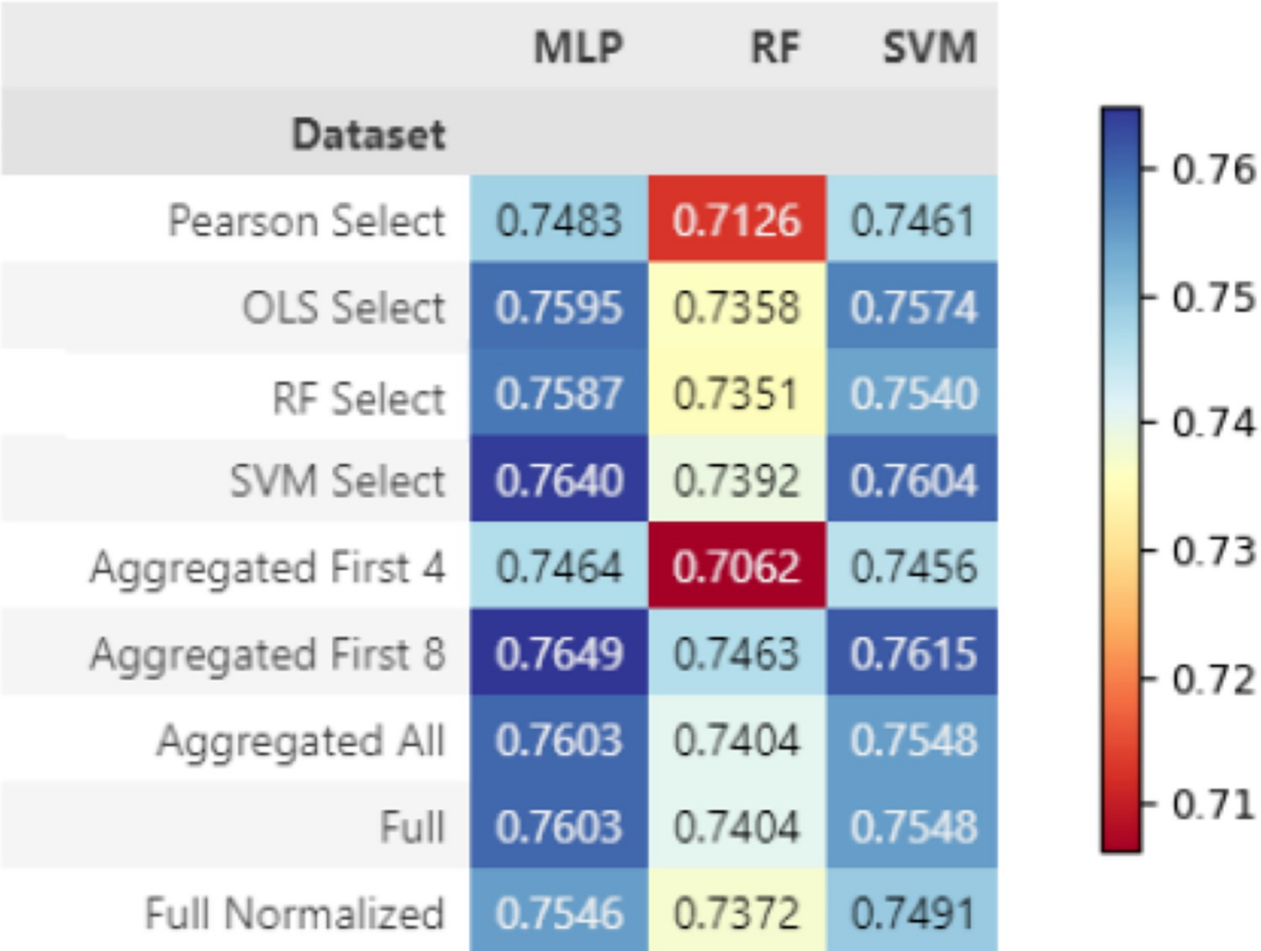
Final sarcopenia classification results shown in AUC across all dataset and model combinations.

The AUC score of sarcopenia classification for each dataset and model combination are shown in Fig 6. Multilayer perceptrons perform best for sarcopenia classification. The highest AUC score was 0.7649, which is held by an MLP trained on the dataset containing the first 8 variables in the aggregated select features dataset. The SVM selected data has worked well for training a MLP on this task, with a similar AUC of 0.7640. The complete performance metrics reported for each test are shown in Table 8 for sarcopenia in S1 File.

**Fig 7:**
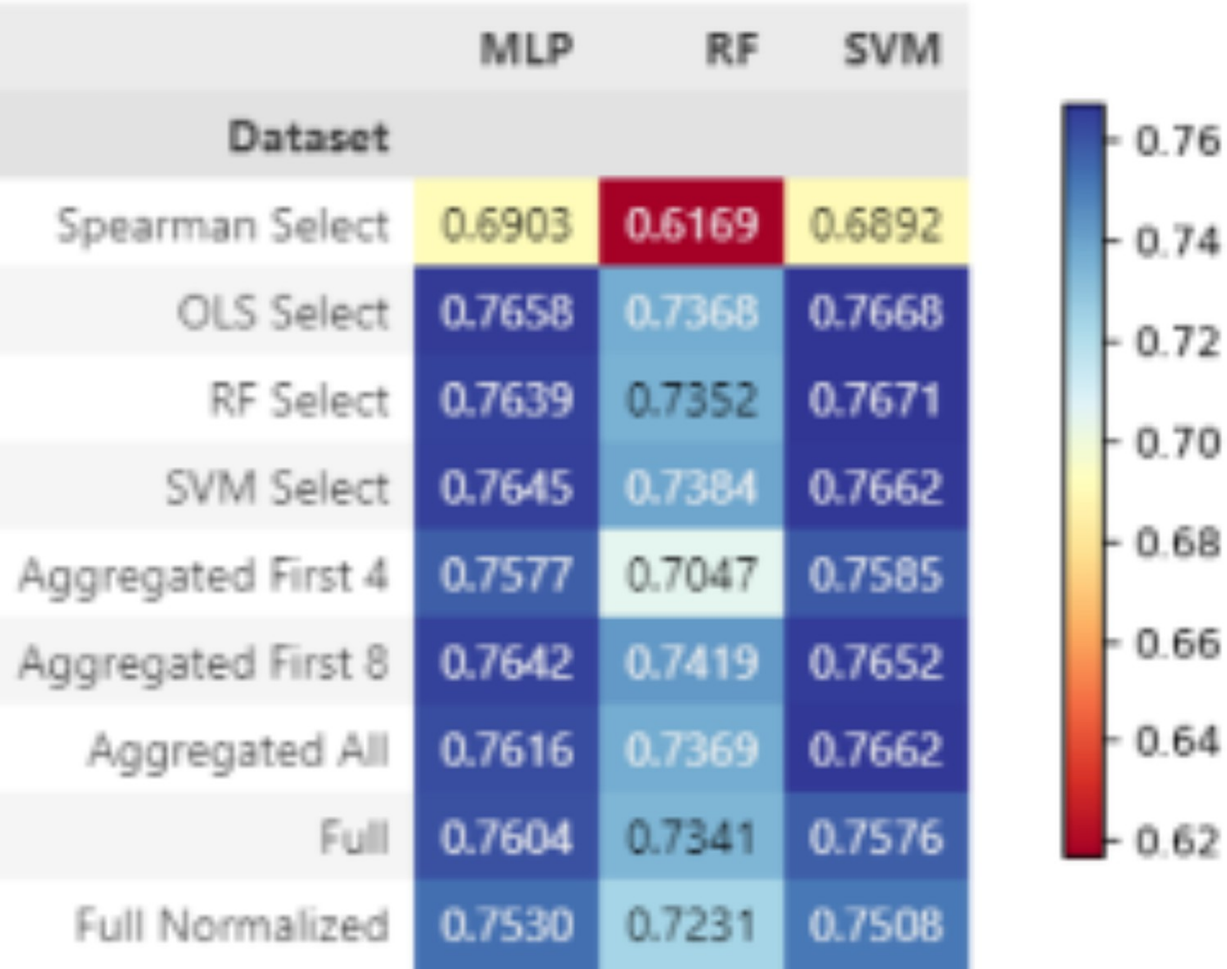
Final classification results shown in AUC across all dataset and model combinations for MQI.

*Final MQI classification results*. The AUC results for each dataset and model combination are shown in Fig 7. The best AUC score achieved for MQI classification was 0.7671, which was given by an SVM trained on the RF selected features. Multilayer perceptrons and SVMs overall perform well at this task with very similar results using all datasets, except for when trained on the Spearman selected dataset. The complete performance metrics reported for each test are shown in Table 9 for MQI in S1 File.

## Discussion

This study aimed to investigate anthropomorphic, functional, and socioeconomic factors associated with muscle quality and sarcopenia, using machine learning approaches. We performed several experiments using feature selection and classification to build predictive models for MQI and sarcopenia and find underlying risk factors. The prediction models showed an AUC performance of 0.7649 for sarcopenia and 0.7671 for MQI, using few features. There was little variation between feature selection methods, highlighting the importance of the features we had originally selected to inspect for both conditions. The explored prediction models have shown a slight increase in predictive power when estimating sarcopenia over MQI. Furthermore, we have demonstrated SVMs and MLPs to have higher overall AUC in predicting each condition. Utilizing an aggregation of feature selection techniques, we were able to investigate the variables most associated with sarcopenia and MQI. For sarcopenia we have found *relative power* and *age* to be the most influential features, with *relative power* being the most significant for predicting muscle quality.

We investigated four approaches to feature selection, which largely agree on selected features. Spearman correlation and SVM methods both use a threshold, which is one cause for the discrepancy present in the features selected. There is not a set rule regarding which threshold should be used for either method. Previous studies using the correlation coefficient make use of different thresholds based on the specific field of research [42, 55]. When using SVM for feature selection there are a variety of approaches to selecting a threshold, including a threshold based on the percentage of features [56], dataset complexity, or other empirically proven threshold values [57]. Determining an appropriate threshold is not a simple task, with too high a threshold possibly removing relevant risk factors for disease and too low including those that are unrelated [58]. One study has suggested automatically selecting a threshold based on model performance [55]. Future work investigating MQI and sarcopenia feature selection could automate the threshold selection process and derive a deeper insight into risk factors for each disease.

From a clinical and data science perspective we believed it was crucial to avoid including pre-established diagnostic variables for feature selection and model training tasks. For our experiments we removed the variables that form part of the clinical diagnostic batteries of each disease, such as *handgrip* and *gait speed (m/s)* in the case of sarcopenia. Incorporating these variables in our dataset could cause both feature selection and model training tasks to replicate the existing diagnostic process rather than helping us to discover other potentially relevant characteristics that could be associated with sarcopenia or MQI. One related study compared sarcopenia prediction amongst different ML algorithms and reported accuracies of 91.7% and upward [59]. However, that study included features such as *handgrip* and *gait speed* in the training data. If we re-run our classification test and add these features to our training data, we also can report higher AUC scores, such as 96.49% when training a RF to predict sarcopenia. However, this does not provide us further insight into understanding sarcopenia. By removing these variables, we were able to investigate underlying and potentially unknown risk factors, thus contributing to a deeper understanding of each disease and improved clinical intervention strategies.

By training prediction models on the different datasets, we were able to further evaluate the importance of each feature for both sarcopenia and MQI. Spearman correlation selected the least number of features for both MQI and sarcopenia; one and three features respectively. With MQI there is marked performance increase when including up to the first four most important features. Performance remains the same as more of the selected variables are used in training, indicating that the variables beyond the first four in the list do not provide significant information. However, prediction is best when using the full, unfiltered dataset. With sarcopenia the performance increases up until and including use of the full dataset. This demonstrates the collective importance of each of these features in our relatively low-dimensional data, where we had already reduced from the original 39 features. The optimal prediction performance for sarcopenia and MQI can be ensured by considering the selected features collectively. In other words, sarcopenia and MQI need to be explained by comprehensively considering all selected features.

### Relevant features for sarcopenia

In our study the variables selected using machine learning that influence sarcopenia, as shown in Fig 1, are the following:

- *Relative power* is the largest contributing factor in the identification of sarcopenia. Sarcopenia is initially identified by low muscle strength and confirmed by diminished muscle quantity or quality. The severity is established when low physical performance is also detected [8]. Muscle power is defined as the result of muscle force multiplied by contraction velocity [60]. Furthermore, it stands out as one of the most dependable indicators of muscle function, showing a robust correlation with functional strength performance among older individuals [13, 60, 61]. Age-related decline in muscle power happens at a faster rate compared to the loss of strength and mass [62, 63], likely due to a decrease in the size of type II fibers or atrophy of the remaining fibers, among other contributing factors [62–64]. Given its tendency to decline early on, muscle power emerges as a feature in the evolution of sarcopenia, as its decline can serve as a key marker of the loss of functionality, muscle mass, and strength associated with this condition. Additional studies corroborate muscle power as a predictor in the assessment of sarcopenia [65, 66].
- *Age* is an influential feature. Sarcopenia, traditionally exclusively associated with the aging process, is now recognized as a multifactorial phenomenon that may manifest in earlier stages of life [8]. However, it remains closely related to aging and its effects, which involve a decline in muscular, neuronal and cognitive function [8, 67]. Beyond the age of 50, a gradual loss of muscle mass (1–2% annually) and strength (1.5–5% annually) is evident [68]. Handgrip strength and walking speed, two dependent variables in the sarcopenia model, also decline with age in both sexes [8, 67], emphasizing the importance of age as a determining factor [13].
- The *5STS* test is also regarded as a feature describing sarcopenia, as the time to complete the test provides insight into strength and locomotor capacity and can be used as a proxy for the strength of leg muscles [8, 69]. As noted above, muscle strength is prioritized as the main parameter for assessing probable sarcopenia within the diagnostic framework, underlining its pivotal role [8, 70].
- *Height*, *weight* and *BMI* are features relevant to sarcopenia. BMI is calculated by dividing a person’s weight in kilograms by the square of their height in meters [71]. It is well recognized that individuals with sarcopenia frequently have lower BMIs, both worldwide [72] and within Spanish populations [73, 74]. In addition, BMI is highlighted in other studies as an important feature in several prediction models [75, 76]. This relationship can be explained by the fact that an increase in BMI, usually accompanied by an increase in both muscle mass and body fat in adults, is associated with lower mortality and a reduced risk of cardiovascular diseases [72, 74]. Furthermore, it may be advantageous to be slightly overweight [77]; while excess body fat has been associated with increased all-cause and disease mortality, people with low, lean body mass have higher mortality rates [72, 74]. As BMI only considers total body mass and not body composition, it may not be a suitable indicator for older people as it does not distinguish between fat and muscle mass [72, 78].
- *Fat mass* is also regarded as a feature describing sarcopenia. In older populations, it may be advantageous to be slightly overweight [77]. However, obesity and excess fat mass exacerbate the risk of sarcopenia, as fat infiltration into the muscle reduces physical function [72, 74]. Additionally, this fat mass is associated with metabolic issues that can affect sarcopenia, and it has differing effects depending on gender. In women, very low percentages of body fat can detrimentally affect muscle status since adipose tissue is an essential endocrine organ that regulates hormonal levels [72, 79]. Excess fat mass also contributes to health conditions, but these impact both genders and entail heightened risks of sarcopenia [72, 80].
- Another influential factor is *sex*, with studies revealing sex-specific etiopathogenic patterns in age-related sarcopenia [61, 81]. Although the diagnostic tools are the same, the cut off points vary between genders [8]. Epidemiological data on the prevalence of sarcopenia among elderly men and women are also contradictory, although many highlight a higher prevalence in men [82, 83]. These differences in risk factors between sexes emphasize the importance of considering sex as a predictive factor when assessing and predicting sarcopenia in older adults.
- *Balance* is another factor influencing sarcopenia. The severity of sarcopenia is determined by physical performance, a multidimensional concept that encompasses not only muscular aspects but also the central and peripheral nervous systems, including balance [8]. Muscle weakness, sarcopenia, and frailty are correlated with an incapacity to maintain balance and results from different studies indicate inferior balance capabilities among those with sarcopenia [84, 85]. Additionally, maintaining balance is crucial to prevent falls, and from a pathophysiological perspective, sarcopenia and decreased balance could increase the risk of falls, thereby worsening the initial pathological condition [85, 86].
- To confirm sarcopenia once the loss of strength has been identified, the loss of *muscle mass* becomes a relevant factor [8]. This progressive age-related process results in substantial declines in both the functional and quantitative aspects of muscle, leading to significant muscle loss [87], and lower muscle mass ratios are linked with sarcopenia [72, 88]. This underscores the importance of preserving muscle mass and strength, given the elevated mortality risk associated with muscle disorders [72, 88]. In addition, there are gender differences that influence the mechanisms of age-related muscle loss; men tend to experience higher absolute rates of muscle loss than women, possibly due to greater initial mass and varied responses to anabolic and catabolic stimuli [89, 90].
- *Socioeconomic index* is also noted as a feature influencing sarcopenia. It is consistent with the literature, as the prevalence of better health and functional outcomes tends to be found in individuals with higher income and education [91, 92].

### Relevant features for MQI

In our study the variables selected using machine learning that influence MQI, as shown in Fig 2, are the following:

- The results highlight the importance of *relative power* in the assessment of MQI. Muscle quality index is defined as the muscular force per unit of muscle mass [35], thus highlighting the functional relevance of muscle architecture and the characteristics of musculoskeletal tissue [93]. The production of maximum force, a key indicator in muscular function, is influenced by morphological factors, muscular architectural features and neural factors [35]. Relative power is an important component of MQI, since it captures the functional importance of the muscular architecture, determining force production capacity and physical function [94]. Several studies find that muscle quality influences muscle power [95, 96] and that reduction in muscle quality is associated with a decrease in strength and power in aged individuals [97]. The loss of muscle power might suggest a decline in muscle quality, as it reflects the organization of neuromuscular factors and the muscle’s ability to produce force, which cannot be fully explained by the reduction in muscle mass alone [35].
- *Fat mass*, *weight and BMI* have also been shown to be important features describing MQI. Obesity characterized by an excessive accumulation of body fat mass increases the infiltration of fat into muscle, lowering physical function and an unfavorable burden on muscle quality [8, 98]. Research has found negative associations between fat mass and obesity with MQI [98, 99]. Other studies have also observed these associations in individuals with pathologies, as higher body fat is linked to MQI deterioration [100]. The same applies to BMI, as elevated BMI has been associated with increased fat infiltration into skeletal muscle. This occurs when BMI is high due to elevated fat mass and decreased muscle mass [100, 101]. However, BMI is not able to distinguish between fat and muscle mass [100, 101]. Additionally, as people age, instead of the loss of muscle mass and strength resulting in weight loss, muscle is often replaced by visceral fat, leading to a preservation of weight but increased muscle weakness [102].
- Another influential feature is the *5STS test*, a commonly employed functional test to assess lower body strength, power, and functional capacity [103, 104]. Although MQI is considered a more comprehensive measure of muscle quality than muscle strength alone, this indirect measure of muscle quality is based on a ratio between overall strength and muscle mass, which may explain why it influences the assessment. Therefore, the 5STS provides valuable information about the lower limb that complements the evaluation of MQI [104, 105]. Additionally, several studies have correlated higher MQ with increased strength, function, and physical performance [94, 106].
- *Gait speed*, also regarded as a feature describing MQI, is seen as a crucial indicator of health and functional condition in elderly individuals [107]. Various walking speed tests are available, with one common option being the 4m gait speed test [108]. Gait speed, indicating physical function and mobility, may be influenced by muscle quality. Good MQ suggests stronger muscles relative to mass, potentially leading to an improvement in walking speed and overall physical performance. This is supported by various studies, which demonstrate that muscle quality significantly impacts walking speed [109, 110]. However, it is worth noting that not all findings align in the same direction, as some articles have found no significant relationship between gait velocity and muscle quality [111].
- *Age* emerges as an influential feature in MQI. The aging process induces neural and morphological alterations in the human musculoskeletal system, leading to a decline in muscular parameters [112]. This reduction in muscular parameters subsequently contributes to the loss of MQI. Various factors contribute to this loss, including compositional changes such as fat infiltration or fibrosis, diminished aerobic capacity, and alterations in metabolism and neural activation [95, 113].
- *Balance* also emerges as an important feature in MQI. However, the literature presents controversy regarding the associations between balance and MQI, tending to link them with dynamic balance and fear of falling rather than static balance [114, 115].

## Conclusion

This research aimed to explore the determinant factors influencing muscle quality and sarcopenia in the older adult population of Bilbao through a machine learning approach. The study encompassed a thorough analysis of participant characteristics, feature selection processes, and classification results to unveil the intricate relationships between various anthropometric, functional, and socioeconomic factors with muscle quality index (MQI) and sarcopenia.

The predictive models used in this study exhibited accuracy rates of 72.78% for MQI and 74.14% for sarcopenia using limited features, with the most successful algorithms being SVM and MLP. This underscores the importance of the features that were used to train the models as well as the effectiveness of machine learning approaches in understanding complex health conditions.

The exploration of feature selection methodologies revealed the collective importance of selected features in predicting both sarcopenia and MQI accurately. Notably, this investigation highlights the pivotal role of features such as *relative power*, *age*, *weight*, and the *5STS test* in predicting both conditions. However, no single factor is sufficient to predict either condition, and by using more features we are able to achieve better predictive results. By comprehensively considering all selected features, the study underscores the importance of a holistic approach in understanding and addressing sarcopenia and MQI among older adults.

The study has several limitations; firstly, the number of patients in the database is small, which limits the ability to obtain results. In addition, the profile of the users is not as diverse as the general world population, as most of the patients are Basque, with similar demographic, racial and body characteristics. This homogeneity in the sample reduces the generalisability of the model, as it does not adequately reflect the variability of a more diverse population, which could lead to biases in the results and limitations in the applicability of the conclusions to a global context.

This research contributes to advancing our understanding of the determinants of muscle quality and sarcopenia, offering valuable insights for developing targeted intervention strategies and improving clinical outcomes in older adult populations. Moving forward, further research endeavors could focus on refining feature selection methodologies, exploring additional factors influencing muscle quality and sarcopenia, and devising tailored interventions to mitigate the burden of these conditions on public health.

## Supporting information

S1 File (DOCX)

S2 File Data. (XLSX)

S3 File Code repository. The repository can be found at https://github.com/dmdequin/sarcopenia_and_machine_learning.
(DOCX)

## References

[1] López-Otín C, Blasco MA, Partridge L, Serrano M, Kroemer G. The hallmarks of aging. Cell. 6 de junio de 2013;153(6):1194–217. doi: 10.1016/j.cell.2013.05.039 23746838 PMC3836174

[2] López-Otín C, Pietrocola F, Roiz-Valle D, Galluzzi L, Kroemer G. Meta-hallmarks of aging and cancer. Cell Metab. 3 de enero de 2023;35(1):12–35. doi: 10.1016/j.cmet.2022.11.001 36599298

[3] Tarekegn A, Ricceri F, Costa G, Ferracin E, Giacobini M. Predictive Modeling for Frailty Conditions in Elderly People: Machine Learning Approaches. JMIR Medical Informatics. 4 de junio de 2020;8(6):e16678. doi: 10.2196/16678 32442149 PMC7303829

[4] Kojima G, Liljas AEM, Iliffe S. Frailty syndrome: implications and challenges for health care policy. Risk Manag Healthc Policy. 2019;12:23–30. doi: 10.2147/RMHP.S168750 30858741 PMC6385767

[5] Sayer AA, Cruz-Jentoft A. Sarcopenia definition, diagnosis and treatment: consensus is growing. Age Ageing. 6 de octubre de 2022;51(10):afac220. doi: 10.1093/ageing/afac220 36273495 PMC9588427

[6] IKEZOE T. Age-Related Change in Muscle Characteristics and Resistance Training for Older Adults. Phys Ther Res. 4 de diciembre de 2020;23(2):99–105. doi: 10.1298/ptr.R0009 33489646 PMC7814211

[7] Reijnierse EM, Trappenburg MC, Blauw GJ, Verlaan S, de van der Schueren MAE, Meskers CGM, et al. Common Ground? The Concordance of Sarcopenia and Frailty Definitions. J Am Med Dir Assoc. 1 de abril de 2016;17(4):371.e7–12. doi: 10.1016/j.jamda.2016.01.013 26922807

[8] Cruz-Jentoft AJ, Bahat G, Bauer J, Boirie Y, Bruyère O, Cederholm T, et al. Sarcopenia: revised European consensus on definition and diagnosis. Age and Ageing. 1 de enero de 2019;48(1):16–31. doi: 10.1093/ageing/afy169 30312372 PMC6322506

[9] McGregor RA, Cameron-Smith D, Poppitt SD. It is not just muscle mass: a review of muscle quality, composition and metabolism during ageing as determinants of muscle function and mobility in later life. Longev Healthspan. 1 de diciembre de 2014;3:9. doi: 10.1186/2046-2395-3-9 25520782 PMC4268803

[10] Goodpaster BH, Park SW, Harris TB, Kritchevsky SB, Nevitt M, Schwartz AV, et al. The Loss of Skeletal Muscle Strength, Mass, and Quality in Older Adults: The Health, Aging and Body Composition Study. The Journals of Gerontology: Series A. 1 de octubre de 2006;61(10):1059–64. 10.1093/gerona/61.10.1059 17077199

[11] Distefano G, Goodpaster BH. Effects of Exercise and Aging on Skeletal Muscle. Cold Spring Harb Perspect Med. 3 de enero de 2018;8(3):a029785. doi: 10.1101/cshperspect.a029785 28432116 PMC5830901

[12] Hortobágyi T, Vetrovsky T, Brach JS, van Haren M, Volesky K, Radaelli R, et al. Effects of Exercise Training on Muscle Quality in Older Individuals: A Systematic Scoping Review with Meta-Analyses. Sports Medicine—Open. 6 de junio de 2023;9(1):41. doi: 10.1186/s40798-023-00585-5 37278947 PMC10244313

[13] Chodzko-Zajko WJ, Proctor DN, Fiatarone Singh MA, Minson CT, Nigg CR, Salem GJ, et al. Exercise and Physical Activity for Older Adults. Medicine & Science in Sports & Exercise. julio de 2009;41(7):1510. 19516148 10.1249/MSS.0b013e3181a0c95c

[14] Choi Y, Kim D, Kim SK. Effects of Physical Activity on Body Composition, Muscle Strength, and Physical Function in Old Age: Bibliometric and Meta-Analyses. Healthcare. enero de 2024;12(2):197. doi: 10.3390/healthcare12020197 38255085 PMC10815094

[15] World Health Organization. (2019). Global action plan on physical activity 2018–2030: More active people for a healthier world. World Health Organization.—Buscar con Google [Internet]. [citado 12 de abril de 2024]. Disponible en: https://www.google.com/search?q=World+Health+Organization.+(2019).+Global+action+plan+on+physical+activity+2018-2030%3A+More+active+people+for+a+healthier+world.+World+Health+Organization.&rlz=1C1GCEA_enES1021ES1021&oq=World+Health+Organization.+(2019).+Global+action+plan+on+physical+activity+2018-2030%3A+More+active+people+for+a+healthier+world.+World+Health+Organization.&gs_lcrp=EgZjaHJvbWUyBggAEEUYOTIGCAEQRRhA0gEHNzg2ajBqN6gCALACAA&sourceid=chrome&ie=UTF-8.

[16] Cesari M, Landi F, Vellas B, Bernabei R, Marzetti E. Sarcopenia and physical frailty: two sides of the same coin. Front Aging Neurosci. 2014;6:192. doi: 10.3389/fnagi.2014.00192 25120482 PMC4112807

[17] Alpaydin Ethem. Introduction to Machine Learning, fourth edition—Ethem Alpaydin—Google Libros [Internet]. 2020 [citado 20 de mayo de 2024]. Disponible en: https://books.google.es/books?hl=es&lr=&id=uZnSDwAAQBAJ&oi=fnd&pg=PR7&ots=xOrXryMlvX&sig=09qydRinPIc7tRHZlpwM5bwcFig#v=onepage&q&f=false.

[18] Bi Q, Goodman KE, Kaminsky J, Lessler J. What is Machine Learning? A Primer for the Epidemiologist. American Journal of Epidemiology. 31 de diciembre de 2019;188(12):2222–39. doi: 10.1093/aje/kwz189 31509183

[19] Kang YJ, Yoo JI, Ha YC. Sarcopenia feature selection and risk prediction using machine learning: A cross-sectional study. Medicine (Baltimore). octubre de 2019;98(43):e17699. doi: 10.1097/MD.0000000000017699 31651901 PMC6824801

[20] Zupo R, Moroni A, Castellana F, Gasparri C, Catino F, Lampignano L, et al. A Machine-Learning Approach to Target Clinical and Biological Features Associated with Sarcopenia: Findings from Northern and Southern Italian Aging Populations. Metabolites. abril de 2023;13(4):565. doi: 10.3390/metabo13040565 37110223 PMC10142879

[21] Sajeev S, Champion S, Maeder A, Gordon S. Machine learning models for identifying pre-frailty in community dwelling older adults. BMC Geriatrics. 12 de octubre de 2022;22(1):794. doi: 10.1186/s12877-022-03475-9 36221059 PMC9554971

[22] Turimov Mustapoevich D, Kim W. Machine Learning Applications in Sarcopenia Detection and Management: A Comprehensive Survey. Healthcare. enero de 2023;11(18):2483. doi: 10.3390/healthcare11182483 37761680 PMC10531485

[23] Suni J., Husu P., & Rinne M. Fitness for Health: The ALPHA-FIT Test Battery for Adults Aged 18–69. Tester’s Manual–ScienceOpen [Internet]. 2009 [citado 29 de abril de 2024]. Disponible en: https://www.scienceopen.com/document?vid=5fa902af-0975-4d8a-8319-b6c5d0ea9d40.

[24] Huang L, Liu Y, Lin T, Hou L, Song Q, Ge N, et al. Reliability and validity of two hand dynamometers when used by community-dwelling adults aged over 50 years. BMC Geriatr. 15 de julio de 2022;22(1):580. 35840905 10.1186/s12877-022-03270-6 PMC9284760

[25] Mezei M, Popescu O, Pricop A, Răchită I. Aspects Of Body Composition In Overweight Students Using Bioelectrical Impedance Measurements. European Proceedings of Social and Behavioural Sciences [Internet]. 16 de febrero de 2019 [citado 29 de abril de 2024];Education and Sports Science in the 21st Century. Disponible en: https://www.europeanproceedings.com/article/10.15405/epsbs.2019.02.85.

[26] Molero Jurado M del M, Pérez Fuentes M del C. Salud y calidad de vida en adultos mayores institucionalizados. International Journal of Developmental and Educational Psychology: INFAD Revista de Psicología. 2011;4(1):249–58.

[27] Domínguez-Berjón MF, Borrell C, Cano-Serral G, Esnaola S, Nolasco A, Pasarín MI, et al. Construcción de un índice de privación a partir de datos censales en grandes ciudades españolas: (Proyecto MEDEA). Gaceta Sanitaria. junio de 2008;22(3):179–87. 10.1157/13123961 18579042

[28] Eustat. Renta personal media de la C.A. de Euskadi por barrio de residencia de las capitales, según tipo de renta (euros). 2021 [Internet]. 2023 [citado 29 de abril de 2024]. Disponible en: https://www.eustat.eus/elementos/ele0006200/renta-personal-media-de-la-ca-de-euskadi-por-barrio-de-residencia-de-las-capitales-segun—tipo-de-renta-euros/tbl0006267_c.html.

[29] Gómez JF, Curcio CL, Alvarado B, Zunzunegui MV, Guralnik J. Validity and reliability of the Short Physical Performance Battery (SPPB): a pilot study on mobility in the Colombian Andes. Colombia Médica: CM. septiembre de 2013;44(3):165. 24892614 PMC4002038

[30] Santamaría-Peláez M, González-Bernal JJ, Da Silva-González Á, Medina-Pascual E, Gentil-Gutiérrez A, Fernández-Solana J, et al. Validity and Reliability of the Short Physical Performance Battery Tool in Institutionalized Spanish Older Adults. Nurs Rep. 30 de septiembre de 2023;13(4):1354–67. doi: 10.3390/nursrep13040114 37873821 PMC10594495

[31] Guralnik JM, Simonsick EM, Ferrucci L, Glynn RJ, Berkman LF, Blazer DG, et al. A short physical performance battery assessing lower extremity function: association with self-reported disability and prediction of mortality and nursing home admission. J Gerontol. marzo de 1994;49(2):M85–94. 10.1093/geronj/49.2.m85 8126356

[32] Ferrari L, Bochicchio G, Bottari A, Lucertini F, Scarton A, Pogliaghi S. Estimating Muscle Power of the Lower Limbs through the 5-Sit-to-Stand Test: A Comparison of Field vs. Laboratory Method. Applied Sciences. enero de 2022;12(22):11577.

[33] Alcazar J, Losa-Reyna J, Rodriguez-Lopez C, Alfaro-Acha A, Rodriguez-Mañas L, Ara I, et al. The sit-to-stand muscle power test: An easy, inexpensive and portable procedure to assess muscle power in older people. Exp Gerontol. 2 de octubre de 2018;112:38–43. doi: 10.1016/j.exger.2018.08.006 30179662

[34] Chang CJ, Lin CH, Hsieh HM, Lo WY, Lai YH, Peng LN, et al. Risk of sarcopenia among older persons with Type 2 diabetes mellitus with different status of albuminuria: A dose-responsive association. Archives of Gerontology and Geriatrics. 1 de julio de 2021;95:104338. doi: 10.1016/j.archger.2021.104338 33652335

[35] Barbat-ArtigasS, RollandY, ZamboniM, Aubertin-LeheudreM. How to assess functional status: A new muscle quality index. The Journal of nutrition, health and aging. 1 de enero de 2012;16(1):67–77. doi: 10.1007/s12603-012-0004-5 22238004

[36] Chandrashekar G, Sahin F. A survey on feature selection methods. Computers & Electrical Engineering. 1 de enero de 2014;40(1):16–28.

[37] Duda RO, Hart PE, Stork DG. Part 1: Pattern Classifcation.

[38] Kupinski MA, Giger ML. Feature selection with limited datasets. Medical Physics. 1999;26(10):2176–82. doi: 10.1118/1.598821 10535635

[39] Saeys Y, Inza I, Larrañaga P. A review of feature selection techniques in bioinformatics. Bioinformatics. 1 de octubre de 2007;23(19):2507–17. doi: 10.1093/bioinformatics/btm344 17720704

[40] Soares I, Dias J, Rocha H, do Carmo Lopes M, Ferreira B. Feature Selection in Small Databases: A Medical-Case Study. En: Kyriacou E, Christofides S, Pattichis CS, editores. XIV Mediterranean Conference on Medical and Biological Engineering and Computing 2016. Cham: Springer International Publishing; 2016. p. 814–9.

[41] Schober P, Boer C, Schwarte LA. Correlation Coefficients: Appropriate Use and Interpretation. Anesthesia & Analgesia. mayo de 2018;126(5):1763. doi: 10.1213/ANE.0000000000002864 29481436

[42] Institut Teknologi Sepuluh Nopember, Sabilla S, Sarno R, Institut Teknologi Sepuluh Nopember, Triyana K, Universitas Gadjah Mada Sekip Utara. Optimizing Threshold using Pearson Correlation for Selecting Features of Electronic Nose Signals. IJIES. 31 de diciembre de 2019;12(6):81–90.

[43] Pilnenskiy N, Smetannikov I. Feature Selection Algorithms as One of the Python Data Analytical Tools. Future Internet. marzo de 2020;12(3):54.

[44] scikit-learn [Internet]. [citado 15 de abril de 2024]. Feature importances with a forest of trees. Disponible en: https://scikit-learn/stable/auto_examples/ensemble/plot_forest_importances.html.

[45] Kaur S, Kalra DS. Feature extraction techniques using support vector machines in disease prediction.

[46] Chauhan NK, Singh K. A Review on Conventional Machine Learning vs Deep Learning. En: 2018 International Conference on Computing, Power and Communication Technologies (GUCON) [Internet]. 2018 [citado 21 de mayo de 2024]. p. 347–52. Disponible en: https://ieeexplore.ieee.org/abstract/document/8675097.

[47] Safonova A, Ghazaryan G, Stiller S, Main-Knorn M, Nendel C, Ryo M. Ten deep learning techniques to address small data problems with remote sensing. International Journal of Applied Earth Observation and Geoinformation. 1 de diciembre de 2023;125:103569.

[48] An C., Park Y. W., Ahn S. S., Han K., Kim H., & Lee S. K. Radiomics machine learning study with a small sample size: Single random training-test set split may lead to unreliable results. PLoS One; 2021.16(8), e0256152. doi: 10.1371/journal.pone.0256152 34383858 PMC8360533

[49] Tohka J., & Van Gils M. Evaluation of machine learning algorithms for health and wellness applications: A tutorial. Computers in Biology and Medicine. 2021;132, 104324. doi: 10.1016/j.compbiomed.2021.104324 33774270

[50] Tischio R. M., & Weiss G. M. Identifying classification algorithms most suitable for imbalanced data. Dept. Comput. Inf. Sci., Fordham Univ., The Bronx, NY, USA, Tech. Rep. 2019.

[51] Grandini M., Bagli E., & Visani G. Metrics for multi-class classification: an overview. arXiv preprint arXiv:2008.05756. 2020.

[52] Géron A. Hands-On Machine Learning with Scikit-Learn, Keras, and TensorFlow. O’Reilly Media, Inc.; 2022. 879 p.

[53] Al-Faiz MZ, Ibrahim AA, Hadi SM. The effect of z-score standardization on binary input due the speed of learning in back-propogation neural network. 2018;1(3).

[54] Schratz P, Muenchow J, Iturritxa E, Richter J, Brenning A. Hyperparameter tuning and performance assessment of statistical and machine-learning algorithms using spatial data. Ecological Modelling. 24 de agosto de 2019;406:109–20.

[55] Sugianela Y, Ahmad T. Pearson Correlation Attribute Evaluation-based Feature Selection for Intrusion Detection System. En: 2020 International Conference on Smart Technology and Applications (ICoSTA) [Internet]. 2020 [citado 7 de mayo de 2024]. p. 1–5. Disponible en: https://ieeexplore.ieee.org/abstract/document/9079263.

[56] Bolón-Canedo V, Sánchez-Maroño N, Alonso-Betanzos A. A review of feature selection methods on synthetic data. Knowl Inf Syst. 1 de marzo de 2013;34(3):483–519.

[57] Seijo-Pardo B, Bolón-Canedo V, Alonso-Betanzos A. Testing Different Ensemble Configurations for Feature Selection. Neural Process Lett. 1 de diciembre de 2017;46(3):857–80.

[58] Akarachantachote N, Chadcham S, Saithanu K. Cutoff threshold of variable importance in projection for variable selection. International Journal of Pure and Applied Mathematics. 17 de julio de 2014;94.

[59] Ozgur S, Altinok YA, Bozkurt D, Saraç ZF, Akçiçek SF. Performance Evaluation of Machine Learning Algorithms for Sarcopenia Diagnosis in Older Adults. Healthcare. enero de 2023;11(19):2699. doi: 10.3390/healthcare11192699 37830737 PMC10572141

[60] American College of Sports Medicine, Chodzko-Zajko WJ, Proctor DN, Fiatarone Singh MA, Minson CT, Nigg CR, et al. American College of Sports Medicine position stand. Exercise and physical activity for older adults. Med Sci Sports Exerc. julio de 2009;41(7):1510–30. doi: 10.1249/MSS.0b013e3181a0c95c 19516148

[61] BahatG, KilicC, ErisS, KaranMA. Power Versus Sarcopenia: Associations with Functionality and Physical Performance Measures. The Journal of nutrition, health and aging. 1 de enero de 2021;25(1):13–7. doi: 10.1007/s12603-020-1544-8 33367457

[62] Tanganelli F, Meinke P, Hofmeister F, Jarmusch S, Baber L, Mehaffey S, et al. Type-2 muscle fiber atrophy is associated with sarcopenia in elderly men with hip fracture. Experimental Gerontology. 1 de febrero de 2021;144:111171. doi: 10.1016/j.exger.2020.111171 33248151

[63] Chodzko-Zajko WJ, Proctor DN, Fiatarone Singh MA, Minson CT, Nigg CR, Salem GJ, et al. Exercise and Physical Activity for Older Adults. Medicine & Science in Sports & Exercise. julio de 2009;41(7):1510–30. 19516148 10.1249/MSS.0b013e3181a0c95c

[64] Miljkovic N, Lim JY, Miljkovic I, Frontera WR. Aging of Skeletal Muscle Fibers. Ann Rehabil Med. abril de 2015;39(2):155–62. doi: 10.5535/arm.2015.39.2.155 25932410 PMC4414960

[65] Gray M, Glenn JM, Binns A. Predicting sarcopenia from functional measures among community-dwelling older adults. AGE. 4 de febrero de 2016;38(1):22. doi: 10.1007/s11357-016-9887-0 26846414 PMC5005883

[66] Jones RL, Paul L, Steultjens MPM, Smith SL. Biomarkers associated with lower limb muscle function in individuals with sarcopenia: a systematic review. Journal of Cachexia, Sarcopenia and Muscle. 2022;13(6):2791–806. doi: 10.1002/jcsm.13064 35977879 PMC9745467

[67] Hortobágyi T, Lesinski M, Gäbler M, VanSwearingen JM, Malatesta D, Granacher U. Effects of Three Types of Exercise Interventions on Healthy Old Adults’ Gait Speed: A Systematic Review and Meta-Analysis. Sports Med. diciembre de 2015;45(12):1627–43. doi: 10.1007/s40279-015-0371-2 26286449 PMC4656792

[68] Keller K, Engelhardt M. Strength and muscle mass loss with aging process. Age and strength loss. Muscles Ligaments Tendons J. 24 de febrero de 2014;3(4):346–50. 24596700 PMC3940510

[69] Tapanya W, Sangkarit N, Amput P, Konsanit S. Lower extremity muscle strength equation of older adults assessed by Five Time Sit to Stand Test (FTSST). Hong Kong Physiother J. junio de 2024;44(01):1–10. doi: 10.1142/S1013702523500099 38577394 PMC10988272

[70] ChewJ, YeoA, YewS, LimJP, TayL, DingYY, et al. Muscle Strength Definitions Matter: Prevalence of Sarcopenia and Predictive Validity for Adverse Outcomes Using the European Working Group on Sarcopenia in Older People 2 (EWGSOP2) Criteria. The Journal of nutrition, health and aging. 1 de junio de 2020;24(6):614–8.10.1007/s12603-020-1371-y32510114

[71] Obesity: preventing and managing the global epidemic. Report of a WHO consultation. World Health Organ Tech Rep Ser. 2000;894:i-xii, 1–253. 11234459

[72] Liu C, Cheng KYK, Tong X, Cheung WH, Chow SKH, Law SW, et al. The role of obesity in sarcopenia and the optimal body composition to prevent against sarcopenia and obesity. Front Endocrinol [Internet]. 1 de marzo de 2023 [citado 15 de mayo de 2024];14. Disponible en: https://www.frontiersin.org/journals/endocrinology/articles/10.3389/fendo.2023.1077255/full. doi: 10.3389/fendo.2023.1077255 36936175 PMC10016224

[73] Gómez-Cabello A, Vicente-Rodríguez G, Maldonado S, Casajus J, Ara I. [Aging and body composition: the sarcopenic obesity in Spain]. Nutrición hospitalaria: organo oficial de la Sociedad Española de Nutrición Parenteral y Enteral. 1 de febrero de 2012;27:22–30. 10.1590/S0212-16112012000100004 22566301

[74] Marcos-Pardo PJ, González-Gálvez N, López-Vivancos A, Espeso-García A, Martínez-Aranda LM, Gea-García GM, et al. Sarcopenia, Diet, Physical Activity and Obesity in European Middle-Aged and Older Adults: The LifeAge Study. Nutrients. 22 de diciembre de 2020;13(1):8. doi: 10.3390/nu13010008 33375058 PMC7822002

[75] Kim J hee. Machine Learning Classifier Models for Predicting Sarcopenia in the Elderly Based on Physical Factors [Internet]. medRxiv; 2023 [citado 15 de mayo de 2024]. p. 2023.05.03.23288546. Disponible en: https://www.medrxiv.org/content/10.1101/2023.05.03.23288546v3. 10.1111/ggi.14895 38744528

[76] Kim SH, Yi CH, Lim J. Risk Factors for Sarcopenia, Sarcopenic Obesity, and Sarcopenia Without Obesity in Older Adults. Physical Therapy Korea. 20 de agosto de 2021;28:177–85.

[77] HanP, ZhaoJ, GuoQ, WangJ, ZhangW, ShenS, et al. Incidence, risk factors, and the protective effect of high body mass index against sarcopenia in suburb-dwelling elderly Chinese populations. J Nutr Health Aging. 1 de diciembre de 2016;20(10):1056–60. doi: 10.1007/s12603-016-0704-3 27925147

[78] Liu C, Wong PY, Chung YL, Chow SKH, Cheung WH, Law SW, et al. Deciphering the “obesity paradox” in the elderly: A systematic review and meta-analysis of sarcopenic obesity. Obesity Reviews. 2023;24(2):e13534. doi: 10.1111/obr.13534 36443946

[79] Oh H, Coburn SB, Matthews CE, Falk RT, LeBlanc ES, Wactawski-Wende J, et al. Anthropometric measures and serum estrogen metabolism in postmenopausal women: the Women’s Health Initiative Observational Study. Breast Cancer Res. 11 de marzo de 2017;19(1):28. doi: 10.1186/s13058-017-0810-0 28284224 PMC5346241

[80] Luo X, Ding H, Broyles A, Warden SJ, Moorthi RN, Imel EA. Using machine learning to detect sarcopenia from electronic health records. DIGITAL HEALTH. 1 de enero de 2023;9:20552076231197098. doi: 10.1177/20552076231197098 37654711 PMC10467215

[81] Liao H, Yang Y, Zeng Y, Qiu Y, Chen Y, Zhu L, et al. Use machine learning to help identify possible sarcopenia cases in maintenance hemodialysis patients. BMC Nephrol. 14 de febrero de 2023;24(1):34. doi: 10.1186/s12882-023-03084-7 36788486 PMC9930261

[82] Soh Y, Won CW. Sex differences in impact of sarcopenia on falls in community-dwelling Korean older adults. BMC Geriatrics. 18 de diciembre de 2021;21(1):716. doi: 10.1186/s12877-021-02688-8 34922482 PMC8684116

[83] Petermann-Rocha F, Balntzi V, Gray SR, Lara J, Ho FK, Pell JP, et al. Global prevalence of sarcopenia and severe sarcopenia: a systematic review and meta-analysis. J Cachexia Sarcopenia Muscle. febrero de 2022;13(1):86–99. doi: 10.1002/jcsm.12783 34816624 PMC8818604

[84] Turimov Mustapoevich D, Kim W. Machine Learning Applications in Sarcopenia Detection and Management: A Comprehensive Survey. Healthcare. enero de 2023;11(18):2483. doi: 10.3390/healthcare11182483 37761680 PMC10531485

[85] Serra-Prat M, Palomera E. Muscle Strength, Sarcopenia and Frailty Associations with Balance and Gait Parameters: A Cross-sectional Study. ejgg. 24 de octubre de 2019;1(2):61–6.

[86] Stuck AK, Basile G, Freystaetter G, de Godoi Rezende Costa Molino C, Lang W, Bischoff-Ferrari HA. Predictive validity of current sarcopenia definitions (EWGSOP2, SDOC, and AWGS2) for clinical outcomes: A scoping review. Journal of Cachexia, Sarcopenia and Muscle. 2023;14(1):71–83. doi: 10.1002/jcsm.13161 36564353 PMC9891988

[87] Rosenberg IH. Sarcopenia: Origins and clinical relevance. Clinics in Geriatric Medicine. 2011;27(3):337–9. doi: 10.1016/j.cger.2011.03.003 21824550

[88] Scheerman K, Meskers CGM, Verlaan S, Maier AB. Sarcopenia, Low Handgrip Strength, and Low Absolute Muscle Mass Predict Long-Term Mortality in Older Hospitalized Patients: An Observational Inception Cohort Study. Journal of the American Medical Directors Association. 1 de abril de 2021;22(4):816–820.e2. doi: 10.1016/j.jamda.2020.12.016 33453174

[89] Tay L, Ding YY, Leung BP, Ismail NH, Yeo A, Yew S, et al. Sex-specific differences in risk factors for sarcopenia amongst community-dwelling older adults. Age (Dordr). diciembre de 2015;37(6):121. doi: 10.1007/s11357-015-9860-3 26607157 PMC5005859

[90] Cruz-Jentoft AJ, Bahat G, Bauer J, Boirie Y, Bruyère O, Cederholm T, et al. Sarcopenia: revised European consensus on definition and diagnosis. Age Ageing. enero de 2019;48(1):16–31. doi: 10.1093/ageing/afy169 30312372 PMC6322506

[91] Shankar A, McMunn A, Steptoe A. Health-related behaviors in older adults relationships with socioeconomic status. Am J Prev Med. enero de 2010;38(1):39–46. doi: 10.1016/j.amepre.2009.08.026 20117555

[92] Noppert GA, Brown CS, Chanti-Ketterl M, Hall KS, Newby LK, Cohen HJ, et al. The Impact of Multiple Dimensions of Socioeconomic Status on Physical Functioning Across the Life Course. Gerontol Geriatr Med. 28 de agosto de 2018;4:2333721418794021. doi: 10.1177/2333721418794021 30186891 PMC6113730

[93] Mangine GT, Stratton MT, Almeda CG, Roberts MD, Esmat TA, VanDusseldorp TA, et al. Physiological differences between advanced CrossFit athletes, recreational CrossFit participants, and physically-active adults. PLOS ONE. 7 de abril de 2020;15(4):e0223548. doi: 10.1371/journal.pone.0223548 32255792 PMC7138313

[94] Naimo MA, Varanoske AN, Hughes JM, Pasiakos SM. Skeletal Muscle Quality: A Biomarker for Assessing Physical Performance Capabilities in Young Populations. Front Physiol. 5 de agosto de 2021;12:706699. doi: 10.3389/fphys.2021.706699 34421645 PMC8376973

[95] IKEZOE T. Age-Related Change in Muscle Characteristics and Resistance Training for Older Adults. Phys Ther Res. 4 de diciembre de 2020;23(2):99–105. doi: 10.1298/ptr.R0009 33489646 PMC7814211

[96] Wilhelm EN, Rech A, Minozzo F, Radaelli R, Botton CE, Pinto RS. Relationship between quadriceps femoris echo intensity, muscle power, and functional capacity of older men. Age (Dordr). junio de 2014;36(3):9625. doi: 10.1007/s11357-014-9625-4 24515898 PMC4082605

[97] Yuan H, Kim M. Meta-Analysis on the Association between Echo Intensity, Muscle Strength, and Physical Function in Older Individuals. Ann Geriatr Med Res. diciembre de 2023;27(4):329–37. doi: 10.4235/agmr.23.0101 37743684 PMC10772333

[98] Neto IV de S, Diniz J de S, Alves VP, Ventura Oliveira AR, Barbosa MP de S, da Silva Prado CR, et al. Field-Based Estimates of Muscle Quality Index Determine Timed-Up-and-Go Test Performance in Obese Older Women. Clin Interv Aging. 22 de febrero de 2023;18:293–303. doi: 10.2147/CIA.S399827 36843630 PMC9949998

[99] Reinders I, Murphy RA, Koster A, Brouwer IA, Visser M, Garcia ME, et al. Muscle Quality and Muscle Fat Infiltration in Relation to Incident Mobility Disability and Gait Speed Decline: the Age, Gene/Environment Susceptibility-Reykjavik Study. The Journals of Gerontology: Series A. 1 de agosto de 2015;70(8):1030–6. 10.1093/gerona/glv016 PMC4506318 25748031

[100] Volpato S, Bianchi L, Lauretani F, Lauretani F, Bandinelli S, Guralnik JM, et al. Role of Muscle Mass and Muscle Quality in the Association Between Diabetes and Gait Speed. Diabetes Care. agosto de 2012;35(8):1672–9. doi: 10.2337/dc11-2202 22596176 PMC3402248

[101] Salmón-Gómez L, Catalán V, Frühbeck G, Gomez-Ambrosi J. Relevance of body composition in phenotyping the obesities. Reviews in Endocrine and Metabolic Disorders. 17 de marzo de 2023;24:1–15. 36928809 10.1007/s11154-023-09796-3 PMC10492885

[102] Chen Y, Lin W, Fu L, Liu H, Jin S, Ye X, et al. Muscle quality index and cardiovascular disease among US population-findings from NHANES 2011–2014. BMC Public Health. 1 de diciembre de 2023;23(1):2388. doi: 10.1186/s12889-023-17303-1 38041010 PMC10691039

[103] Jones CJ, Rikli RE, Beam WC. A 30-s chair-stand test as a measure of lower body strength in community-residing older adults. Res Q Exerc Sport. junio de 1999;70(2):113–9. doi: 10.1080/02701367.1999.10608028 10380242

[104] Yee XS, Ng YS, Allen JC, Latib A, Tay EL, Abu Bakar HM, et al. Performance on sit-to-stand tests in relation to measures of functional fitness and sarcopenia diagnosis in community-dwelling older adults. European Review of Aging and Physical Activity. 8 de enero de 2021;18(1):1. doi: 10.1186/s11556-020-00255-5 33419399 PMC7791746

[105] Jerez-Mayorga D, Chirosa Ríos LJ, Reyes A, Delgado-Floody P, Machado Payer R, Guisado Requena IM. Muscle quality index and isometric strength in older adults with hip osteoarthritis. PeerJ. 7 de agosto de 2019;7:e7471. doi: 10.7717/peerj.7471 31410316 PMC6689221

[106] Fragala MS, Jajtner AR, Beyer KS, Townsend JR, Emerson NS, Scanlon TC, et al. Biomarkers of muscle quality: N-terminal propeptide of type III procollagen and C-terminal agrin fragment responses to resistance exercise training in older adults. J Cachexia Sarcopenia Muscle. junio de 2014;5(2):139–48. doi: 10.1007/s13539-013-0120-z 24197815 PMC4053565

[107] Middleton A, Fritz SL, Lusardi M. Walking speed: the functional vital sign. J Aging Phys Act. abril de 2015;23(2):314–22. doi: 10.1123/japa.2013-0236 24812254 PMC4254896

[108] Hirabayashi R, Takahashi Y, Nagata K, Morimoto T, Wakata K, Nakagawa A, et al. The validity and reliability of four-meter gait speed test for stable interstitial lung disease patients: the prospective study. J Thorac Dis. abril de 2020;12(4):1296–304. doi: 10.21037/jtd.2020.02.57 32395266 PMC7212132

[109] Hirano Y, Yamada Y, Matsui Y, Ota S, Arai H. Lower limb muscle quality and phase angle contribute to the reduced walking speed among older adults. Geriatr Gerontol Int. agosto de 2022;22(8):603–9. doi: 10.1111/ggi.14423 35781752

[110] Lin YH, Chen HC, Hsu NW, Chou P. Using hand grip strength to detect slow walking speed in older adults: the Yilan study. BMC Geriatr. 16 de julio de 2021;21:428. doi: 10.1186/s12877-021-02361-0 34271880 PMC8285830

[111] Martinikorena I, Martínez-Ramírez A, Gómez M, Lecumberri P, Casas-Herrero A, Cadore EL, et al. Gait Variability Related to Muscle Quality and Muscle Power Output in Frail Nonagenarian Older Adults. Journal of the American Medical Directors Association. 1 de febrero de 2016;17(2):162–7. doi: 10.1016/j.jamda.2015.09.015 26577625

[112] Goodpaster BH, Park SW, Harris TB, Kritchevsky SB, Nevitt M, Schwartz AV, et al. The Loss of Skeletal Muscle Strength, Mass, and Quality in Older Adults: The Health, Aging and Body Composition Study. The Journals of Gerontology: Series A. 1 de octubre de 2006;61(10):1059–64. doi: 10.1093/gerona/61.10.1059 17077199

[113] McGregor RA, Cameron-Smith D, Poppitt SD. It is not just muscle mass: a review of muscle quality, composition and metabolism during ageing as determinants of muscle function and mobility in later life. Longev Healthspan. 1 de diciembre de 2014;3(1):9. doi: 10.1186/2046-2395-3-9 25520782 PMC4268803

[114] Gadelha AB, Neri SGR, Nóbrega OT, Pereira JC, Bottaro M, Fonsêca A, et al. Muscle quality is associated with dynamic balance, fear of falling, and falls in older women. Experimental Gerontology. 1 de abril de 2018;104:1–6. doi: 10.1016/j.exger.2018.01.003 29329971

[115] Hairi NN, Cumming RG, Naganathan V, Handelsman DJ, Le Couteur DG, Creasey H, et al. Loss of muscle strength, mass (sarcopenia), and quality (specific force) and its relationship with functional limitation and physical disability: the Concord Health and Ageing in Men Project. J Am Geriatr Soc. noviembre de 2010;58(11):2055–62. doi: 10.1111/j.1532-5415.2010.03145.x 21054284

