# Supplementary material for: Exploring determinant factors influencing muscle quality and sarcopenia in Bilbao’s older adult population through machine learning: A comprehensive analysis approach
Source: PLoS One. 2024 Dec 31;19(12):e0316174. doi: 10.1371/journal.pone.0316174 (PMC11687929; doi:10.1371/journal.pone.0316174)
Supplement: S1 File — (DOCX) [file pone.0316174.s001.docx]

**Supporting information:**

**S1.1 Supporting information 1: Parameters testing for machine learning**

Parameters tested for each model during hyperparameter tuning.

**S1.1 Table 1.** Hyperparameters tested for each model during hyperparameter tuning. This table shows each hyperparameter, the option used in the baseline test, and the options tested when searching for the best.

| **Model** | **Hyperparameter** | **Baseline** | **Options Tested** |
| --- | --- | --- | --- |
| SVM | C  kernel  degree  class_weight | 1.0  ‘linear’  3  None | 1.0, 10.0, 100.0  ‘rbf’, ‘linear’, ‘poly’, ‘sigmoid’  3, 4, 5, 6  None, ‘balanced’ |
| MLP | activation  learning_rate  hidden_layer_sizes | ‘relu’  ‘constant’  (100,) | ‘logistic’, ‘tanh’, ‘relu’  ‘constant’, ‘adaptive’  (10,), (100,), (200,), (10,10), (10,100), (100,100), (10,10,10), (100,100,100) |
| RF | n_estimators  max_features  class_weight | 100  ‘sqrt’  None | 1, 2, 4, 8, 16, 32, 64, 100, 200  ‘sqrt’, ‘log2’, None  ‘balanced’, None |

**Supporting information 1.2: Optimal Hyperparameters**

The parameters found to be the best for each dataset during hyperparameter tuning.

**S1. 2 Table 2**: Optimal hyperparameters for each dataset within Sarcopenia.

|  | **MLP** | **RF** | **SVM** |
| --- | --- | --- | --- |
| Spearman Selected | activation: ‘tanh’, hidden_layer_sizes: (10,100),  learning_rate: ‘constant’ | class_weight: None, max_features: ‘sqrt’,  n_estimators: 200 | C: 1.0,  class_weight: None, degree: 3, kernel: ‘rbf’ |
| OLS Selected | activation: ‘logistic’, hidden_layer_sizes: (100,100),  learning_rate: ‘constant’ | class_weight: ‘balanced’, max_features: ‘sqrt’,  n_estimators: 64 | C: 1.0,  class_weight: None, degree: 3, kernel: ‘rbf’ |
| RF Selected | activation: ‘logistic’, hidden_layer_sizes: (10,),  learning_rate: ‘constant’ | class_weight: ‘balanced’, max_features: ‘sqrt’,  n_estimators: 200 | C: 1.0,  class_weight: None, degree: 3, kernel: ‘linear’ |
| SVM Selected | activation: ‘logistic’, hidden_layer_sizes:(10,10),  learning_rate: ‘constant’ | class_weight: None, max_features: ‘sqrt’,  n_estimators: 100 | C: 1.0,  class_weight: None, degree: 3, kernel: ‘rbf’ |
| Aggregated, First 4 | activation: ‘logistic’, hidden_layer_sizes: (100,100,100),  learning_rate: ‘constant’ | class_weight: None, max_features: ‘sqrt’,  n_estimators: 200 | C: 10.0,  class_weight: None, degree: 3, kernel: ‘linear’ |
| Aggregated, First 8 | activation: ‘tanh’, hidden_layer_sizes: (10,100),  learning_rate: ‘constant’ | class_weight: None, max_features: ‘sqrt’,  n_estimators: 100 | C: 1.0,  class_weight: None, degree: 3, kernel: ‘rbf’ |
| Aggregated, All | activation: ‘logistic’, hidden_layer_sizes: (10,),  learning_rate: ‘constant’ | class_weight: ‘balanced’, max_features: ‘sqrt’,  n_estimators: 200 | C: 1.0,  class_weight: None, degree: 3, kernel: ‘rbf’ |
| Full | activation: ‘logistic’, hidden_layer_sizes: (10,),  learning_rate: ‘constant’ | class_weight: ‘balanced’, max_features: ‘sqrt’,  n_estimators: 200 | C: 1.0,  class_weight: None, degree: 3, kernel: ‘rbf’ |
| Full, Normalized | activation: ‘logistic’, hidden_layer_sizes: (10,100),  learning_rate: ‘constant’ | class_weight: ‘balanced’, max_features: ‘sqrt’,  n_estimators: 64 | C: 1.0,  class_weight: None, degree: 3, kernel: ‘linear’ |

**S1. 2 Table 3:** Optimal hyperparameters for each dataset within MQI.

|  | **MLP** | **RF** | **SVM** |
| --- | --- | --- | --- |
| Spearman Selected | activation: ‘relu’, hidden_layer_sizes: (10,10),  learning_rate: ‘constant’ | class_weight: None, max_features: ‘sqrt’,  n_estimators: 64 | C: 1.0, class_weight: None, degree: 3, kernel: ‘rbf’ |
| OLS Selected | activation: ‘relu’, hidden_layer_sizes: (10,100),  learning_rate: ‘constant’ | class_weight: None, max_features: None,  n_estimators: 200 | C: 1.0, class_weight: None, degree: 3, kernel: ‘linear’ |
| RF Selected | activation: ‘relu’, hidden_layer_sizes: (10,10),  learning_rate: ‘constant’ | class_weight: None, max_features: ‘sqrt’,  n_estimators: 100 | C: 1.0, class_weight: None, degree: 3, kernel: ‘linear’ |
| SVM Selected | activation: ‘logistic’, hidden_layer_sizes: (10,10),  learning_rate: ‘constant’ | class_weight: None, max_features: ‘sqrt’,  n_estimators: 200 | C: 10.0, class_weight: None, degree: 3, kernel: ‘linear’ |
| Aggregated, First 4 | activation: ‘relu’, hidden_layer_sizes: (10,),  learning_rate: ‘constant’ | class_weight: ‘balanced’, max_features: ‘sqrt’,  n_estimators: 64 | C: 10.0, class_weight: None, degree: 3, kernel: ‘linear’ |
| Aggregated, First 8 | activation: ‘relu’, hidden_layer_sizes: (10,),  learning_rate: ‘constant’ | class_weight: None, max_features: ‘sqrt’,  n_estimators: 64 | C: 1.0, class_weight: None, degree: 3, kernel: ‘linear’ |
| Aggregated, All | activation: ‘relu’, hidden_layer_sizes: (200,),  learning_rate: ‘constant’ | class_weight: ‘balanced’, max_features: ‘sqrt’,  n_estimators: 200 | C: 10.0, class_weight: None, degree: 3, kernel: ‘linear’ |
| Full | activation: ‘logistic’, hidden_layer_sizes: (10,10),  learning_rate: ‘constant’ | class_weight: ‘balanced’, max_features: ‘sqrt’,  n_estimators: 200 | C: 10.0, class_weight: None, degree: 3, kernel: ‘linear’ |
| Full, Normalized | activation: ‘logistic’, hidden_layer_sizes: (100,),  learning_rate: ‘constant’ | class_weight: ‘balanced’, max_features: ‘sqrt’,  n_estimators: 200 | C: 1.0, class_weight: None, degree: 3, kernel: ‘linear’ |

**Supporting information 1. 3: Features Per Dataset**

The features present in each dataset for classification tests.

**S1. 3 Table 4**. Features in MQI datasets.

|  | **Spearman** | **OLS** | **RF** | **SVM** | **Aggregated (4)** | **Aggregated (8)** | **Aggregated (Full)** | **Full** | **Full, normalized** |
| --- | --- | --- | --- | --- | --- | --- | --- | --- | --- |
| Socioeconomic Index (3) |  |  |  |  |  |  |  | X | X |
| Age |  | X | X | X |  | X | X | X | X |
| Weight |  | X | X | X | X | X | X | X | X |
| BMI |  | X | X | X | X | X | X | X | X |
| Fat Mass |  | X | X | X | X | X | X | X | X |
| Balance 1 |  | X |  |  |  |  | X | X | X |
| Balance 2 |  |  |  | X |  |  | X | X | X |
| Balance 3 |  |  |  |  |  |  |  | X | X |
| Gait Speed (m/s) |  | X | X | X |  | X | X | X | X |
| 4m Test |  | X | X |  |  |  | X | X | X |
| 5STS |  | X | X | X |  | X | X | X | X |
| Mean Power |  |  | X | X |  | X | X | X | X |
| Relative Power | X | X | X | X | X | X | X | X | X |
| SPPB |  |  |  | X |  |  | X | X | X |
| Frailty |  |  |  |  |  |  |  | X | X |
| Groups |  |  |  | X |  |  | X | X | X |
| Sarcopenia v |  |  |  |  |  |  |  | X | X |

**S1. 3 Table 5**. Features in Sarcopenia datasets.

|  | **Spearman** | **OLS** | **RF** | **SVM** | **Aggregated (4)** | **Aggregated (8)** | **Aggregated (Full)** | **Full** | **Full, normalized** |
| --- | --- | --- | --- | --- | --- | --- | --- | --- | --- |
| Socioeconomic Index (3) |  | X |  |  |  |  | X | X | X |
| Sex |  | X |  | X |  |  | X | X | X |
| Age | X | X | X | X | X | X | X | X | X |
| Height |  | X | X | X | X | X | X | X | X |
| Weight |  | X | X |  |  | X | X | X | X |
| BMI |  | X |  |  |  |  | X | X | X |
| Fat Mass |  |  | X |  |  |  | X | X | X |
| Muscle |  |  | X | X |  | X | X | X | X |
| 5STS | X |  | X | X | X | X | X | X | X |
| Relative Power | X | X | X | X | X | X | X | X | X |
| Points Balance |  | X |  | X |  | X | X | X | X |

**S1. 4 Table 6**. Baseline classification results for Sarcopenia datasets. Best AUC score per model is in bold.

| **Dataset** | **Metric** | **SVM** | **NB** | **KNN** | **RF** | **MLP** | **GB** | **DT** | **SGD** |
| --- | --- | --- | --- | --- | --- | --- | --- | --- | --- |
| **Spearman** | Accuracy | 0,5962 | 0,5923 | 0,526 | 0,5755 | 0,5802 | 0,577 | 0,4877 | 0,5818 |
|  | F1 | 0,4217 | 0,5166 | 0,4395 | 0,5002 | 0,4399 | 0,4842 | 0,4367 | 0,4118 |
|  | AUC | 0,7369 | 0,7446 | 0,6512 | 0,7076 | 0,7418 | 0,714 | 0,5807 | 0,7255 |
| **OLS** | Accuracy | 0,6178 | 0,5372 | 0,522 | 0,5683 | 0,6058 | 0,581 | 0,5068 | 0,5946 |
|  | F1 | 0,437 | 0,4998 | 0,4319 | 0,4864 | 0,4807 | 0,512 | 0,4489 | 0,4289 |
|  | AUC | 0,7552 | 0,7449 | 0,6456 | 0,7359 | 0,755 | **0,7387** | 0,5883 | 0,7389 |
| **RF** | Accuracy | 0,601 | 0,593 | 0,5204 | 0,5962 | 0,589 | 0,5683 | 0,4925 | 0,5954 |
|  | F1 | 0,4248 | 0,5404 | 0,4453 | 0,5237 | 0,4908 | 0,514 | 0,4323 | 0,4321 |
|  | AUC | 0,7476 | **0,751** | 0,6521 | 0,7369 | 0,7495 | 0,7223 | 0,5775 | 0,7392 |
| **PCA** | Accuracy | 0,5962 | 0,5762 | 0,431 | 0,5482 | 0,5778 | 0,5244 | 0,474 | 0,494 |
|  | F1 | 0,4196 | 0,4631 | 0,2636 | 0,4581 | 0,4466 | 0,4536 | 0,4194 | 0,364 |
|  | AUC | 0,7392 | 0,7209 | 0,5238 | 0,7056 | 0,7303 | 0,7158 | 0,5624 | 0,7347 |
| **Full** | Accuracy | 0,6122 | 0,5316 | 0,5435 | 0,5834 | 0,5986 | 0,573 | 0,5036 | 0,6058 |
|  | F1 | 0,4327 | 0,5041 | 0,461 | 0,5077 | 0,489 | 0,5121 | 0,4501 | 0,4429 |
|  | AUC | 0,7508 | 0,7467 | 0,6664 | 0,7376 | 0,7528 | 0,7382 | 0,5908 | 0,7462 |
| **Full normalized** | Accuracy | 0,6122 | 0,5555 | 0,5619 | 0,5978 | 0,5946 | 0,5874 | 0,5005 | 0,601 |
|  | F1 | 0,4335 | 0,5256 | 0,4855 | 0,5 | 0,478 | 0,4898 | 0,4413 | 0,4455 |
|  | AUC | 0,7525 | 0,7468 | **0,688** | **0,7457** | 0,7508 | 0,7296 | 0,5843 | **0,7564** |
| **Aggregated (4)** | Accuracy | 0,5898 | 0,5778 | 0,4908 | 0,5603 | 0,581 | 0,5611 | 0,5108 | 0,589 |
|  | F1 | 0,4164 | 0,5303 | 0,4167 | 0,4822 | 0,5012 | 0,4758 | 0,4491 | 0,4394 |
|  | AUC | 0,7038 | 0,7439 | 0,6341 | 0,705 | 0,7402 | 0,7152 | 0,5885 | 0,724 |
| **Aggregated (8)** | Accuracy | 0,6138 | 0,5452 | 0,5252 | 0,5818 | 0,6042 | 0,5898 | 0,5228 | 0,6042 |
|  | F1 | 0,4336 | 0,5198 | 0,4409 | 0,5024 | 0,4907 | 0,509 | 0,4642 | 0,4405 |
|  | AUC | **0,7585** | 0,7477 | 0,6514 | 0,7444 | 0,7546 | 0,7355 | **0,5995** | 0,7405 |
| **Aggregated (full)** | Accuracy | 0,6122 | 0,5316 | 0,5435 | 0,5834 | 0,5986 | 0,573 | 0,5036 | 0,6058 |
|  | F1 | 0,4327 | 0,5041 | 0,461 | 0,5077 | 0,489 | 0,5121 | 0,4501 | 0,4429 |
|  | AUC | 0,7508 | 0,7467 | 0,6664 | 0,7376 | 0,7528 | 0,7382 | 0,5908 | 0,7462 |
| **SVM** | Accuracy | 0,6138 | 0,5428 | 0,5404 | 0,5779 | 0,5962 | 0,5779 | 0,4988 | 0,6058 |
|  | F1 | 0,4334 | 0,5162 | 0,4657 | 0,4883 | 0,4976 | 0,4823 | 0,4388 | 0,4456 |
|  | AUC | 0,7575 | 0,7468 | 0,6606 | 0,7378 | **0,7582** | 0,7332 | 0,5821 | 0,7459 |

**S1. 4 Table 7**. Baseline classification results for MQI datasets. Best AUC score per model is in bold.

| **Dataset** | **Metric** | **SVM** | **NB** | **KNN** | **RF** | **MLP** | **GB** | **DT** | **SGD** |
| --- | --- | --- | --- | --- | --- | --- | --- | --- | --- |
| **Spearman** | Accuracy | 0,723 | 0,6672 | 0,664 | 0,7127 | 0,7246 | 0,7023 | 0,6025 | 0,7286 |
|  | F1 | 0,4538 | 0,4582 | 0,4724 | 0,4932 | 0,4815 | 0,483 | 0,4465 | 0,4658 |
|  | AUC | 0,7189 | 0,7084 | **0,6718** | 0,7311 | **0,7664** | 0,7377 | 0,5937 | 0,7553 |
| **OLS** | Accuracy | 0,668 | 0,6759 | 0,6049 | 0,6456 | 0,6664 | 0,6624 | 0,6169 | 0,6799 |
|  | F1 | 0,2669 | 0,3653 | 0,3933 | 0,4086 | 0,3158 | 0,3652 | 0,4062 | 0,3597 |
|  | AUC | 0,5306 | 0,6859 | 0,6091 | 0,6156 | 0,6828 | 0,6646 | 0,5926 | 0,6856 |
| **RF** | Accuracy | 0,723 | 0,668 | 0,6584 | 0,7103 | 0,7191 | 0,6991 | 0,5938 | 0,7159 |
|  | F1 | 0,4526 | 0,4651 | 0,4847 | 0,4981 | 0,4696 | 0,4688 | 0,4366 | 0,4452 |
|  | AUC | 0,726 | 0,706 | 0,6644 | 0,7352 | 0,7649 | 0,7378 | 0,585 | 0,7554 |
| **PCA** | Accuracy | 0,7254 | 0,2777 | 0,656 | 0,7087 | 0,7183 | 0,7015 | 0,6097 | 0,723 |
|  | F1 | 0,4608 | 0,2124 | 0,4675 | 0,4796 | 0,4769 | 0,466 | 0,4624 | 0,4549 |
|  | AUC | 0,728 | 0,6698 | 0,6655 | 0,7297 | 0,757 | 0,732 | **0,608** | 0,7492 |
| **Full** | Accuracy | 0,7238 | 0,5187 | 0,6568 | 0,7063 | 0,7119 | 0,7007 | 0,5898 | 0,7191 |
|  | F1 | 0,4637 | 0,4529 | 0,4574 | 0,4875 | 0,4599 | 0,4914 | 0,4469 | 0,4537 |
|  | AUC | 0,683 | 0,683 | 0,6631 | 0,7322 | 0,7488 | 0,7332 | 0,5953 | 0,7491 |
| **Full normalized** | Accuracy | 0,6672 | 0,6807 | 0,5157 | 0,5508 | 0,6831 | 0,53 | 0,4917 | 0,66 |
|  | F1 | 0,2698 | 0,3719 | 0,3676 | 0,3677 | 0,3691 | 0,3484 | 0,3684 | 0,3695 |
|  | AUC | 0,6091 | 0,6792 | 0,5786 | 0,6401 | 0,691 | 0,6543 | 0,5556 | 0,6577 |
| **Aggregated (4)** | Accuracy | 0,7254 | 0,6991 | 0,6592 | 0,6911 | 0,7199 | 0,7015 | 0,581 | 0,7223 |
|  | F1 | 0,4567 | 0,4351 | 0,4795 | 0,4769 | 0,4678 | 0,4779 | 0,4391 | 0,4583 |
|  | AUC | **0,7289** | 0,7122 | 0,65 | 0,6994 | 0,7553 | 0,7259 | 0,5883 | 0,7501 |
| **Aggregated (8)** | Accuracy | 0,7246 | 0,6799 | 0,6624 | 0,7143 | 0,723 | 0,7031 | 0,585 | 0,7135 |
|  | F1 | 0,4548 | 0,4762 | 0,4859 | 0,4994 | 0,47 | 0,4767 | 0,4333 | 0,4414 |
|  | AUC | 0,7231 | **0,7134** | 0,6644 | 0,7374 | 0,7572 | **0,7442** | 0,5847 | 0,7538 |
| **Aggregated (full)** | Accuracy | 0,7222 | 0,1844 | 0,6536 | 0,7159 | 0,719 | 0,7063 | 0,593 | 0,7174 |
|  | F1 | 0,4545 | 0,1242 | 0,4719 | 0,496 | 0,4882 | 0,4776 | 0,4457 | 0,4481 |
|  | AUC | 0,7281 | 0,7018 | 0,6579 | **0,7401** | 0,7561 | 0,736 | 0,5941 | **0,7569** |
| **SVM** | Accuracy | 0,7222 | 0,1844 | 0,6536 | 0,7079 | 0,7127 | 0,7055 | 0,6121 | 0,7198 |
|  | F1 | 0,4545 | 0,1242 | 0,4719 | 0,4816 | 0,4998 | 0,4787 | 0,4597 | 0,4501 |
|  | AUC | 0,7281 | 0,7018 | 0,6579 | 0,7363 | 0,7603 | 0,7372 | 0,6041 | 0,7563 |

**S1. 5 Table 8**. Final classification results for Sarcopenia datasets. Best AUC score per model is in bold.

| **Dataset** | **Metric** | **SVM** | **RF** | **MLP** |
| --- | --- | --- | --- | --- |
| **Spearman** | Accuracy | 0,7461 | 0,7126 | 0,7483 |
|  | F1 | 0,5011 | 0,4896 | 0,5084 |
|  | AUC | 0,7461 | 0,7126 | 0,7483 |
| **OLS** | Accuracy | 0,7574 | 0,7358 | 0,7595 |
|  | F1 | 0,5153 | 0,4906 | 0,5045 |
|  | AUC | 0,7574 | 0,7358 | 0,7595 |
| **RF** | Accuracy | 0,754 | 0,7351 | 0,7587 |
|  | F1 | 0,5042 | 0,5066 | 0,4851 |
|  | AUC | 0,754 | 0,7351 | 0,7587 |
| **Full** | Accuracy | 0,7548 | 0,7404 | 0,7603 |
|  | F1 | 0,4986 | 0,5155 | 0,4723 |
|  | AUC | 0,7548 | 0,7404 | 0,7603 |
| **Full normalized** | Accuracy | 0,7491 | 0,7372 | 0,7546 |
|  | F1 | 0,4283 | 0,4875 | 0,429 |
|  | AUC | 0,7491 | 0,7372 | 0,7546 |
| **Aggregated (4)** | Accuracy | 0,7456 | 0,7062 | 0,7464 |
|  | F1 | 0,5003 | 0,4989 | 0,4554 |
|  | AUC | 0,7456 | 0,7062 | 0,7464 |
| **Aggregated (8)** | Accuracy | 0,7615 | 0,7463 | 0,7649 |
|  | F1 | 0,5045 | 0,515 | 0,4765 |
|  | AUC | **0,7615** | **0,7463** | **0,7649** |
| **Aggregated (full)** | Accuracy | 0,7548 | 0,7404 | 0,7603 |
|  | F1 | 0,4986 | 0,5155 | 0,4723 |
|  | AUC | 0,7548 | 0,7404 | 0,7603 |
| **SVM** | Accuracy | 0,7604 | 0,7392 | 0,764 |
|  | F1 | 0,5058 | 0,4905 | 0,4326 |
|  | AUC | 0,7604 | 0,7392 | 0,764 |

**S1. 5 Table 9**. Final classification results for MQI datasets. Best AUC score per model is in bold.

| **Dataset** | **Metric** | **SVM** | **RF** | **MLP** |
| --- | --- | --- | --- | --- |
| **Spearman** | Accuracy | 0,7668 | 0,7368 | 0,7658 |
|  | F1 | 0,5357 | 0,4901 | 0,4848 |
|  | AUC | **0,7668** | 0,7368 | **0,7658** |
| **OLS** | Accuracy | 0,6892 | 0,6169 | 0,6903 |
|  | F1 | 0,3787 | 0,3911 | 0,3617 |
|  | AUC | 0,6892 | 0,6169 | 0,6903 |
| **RF** | Accuracy | 0,7671 | 0,7352 | 0,7639 |
|  | F1 | 0,5383 | 0,4955 | 0,4771 |
|  | AUC | 0,7671 | 0,7352 | 0,7639 |
| **Full** | Accuracy | 0,7576 | 0,7341 | 0,7604 |
|  | F1 | 0,5328 | 0,465 | 0,4674 |
|  | AUC | 0,7576 | 0,7341 | 0,7604 |
| **Full normalized** | Accuracy | 0,7508 | 0,7232 | 0,753 |
|  | F1 | 0,5291 | 0,4732 | 0,479 |
|  | AUC | 0,7508 | 0,7232 | 0,753 |
| **Aggregated (4)** | Accuracy | 0,7585 | 0,7047 | 0,7577 |
|  | F1 | 0,542 | 0,4664 | 0,4566 |
|  | AUC | 0,7585 | 0,7047 | 0,7577 |
| **Aggregated (8)** | Accuracy | 0,7652 | 0,7419 | 0,7642 |
|  | F1 | 0,5388 | 0,4787 | 0,4707 |
|  | AUC | 0,7652 | **0,7419** | 0,7642 |
| **Aggregated (full)** | Accuracy | 0,7662 | 0,7369 | 0,7616 |
|  | F1 | 0,5466 | 0,4898 | 0,4691 |
|  | AUC | 0,7662 | 0,7369 | 0,7616 |
| **SVM** | Accuracy | 0,7662 | 0,7384 | 0,7645 |
|  | F1 | 0,5466 | 0,4988 | 0,4575 |
|  | AUC | 0,7662 | 0,7384 | 0,7645 |
