# Supplementary material for: Exploring determinant factors influencing muscle quality and sarcopenia in Bilbao’s older adult population through machine learning: A comprehensive analysis approach
Source: PLoS One. 2024 Dec 31;19(12):e0316174. doi: 10.1371/journal.pone.0316174 (PMC11687929; doi:10.1371/journal.pone.0316174)
Supplement: S3 File — The repository can be found at https://github.com/dmdequin/sarcopenia_and_machine_learning. (DOCX) [file pone.0316174.s003.docx]

**S3 File. Code Repository**

The repository can be found at: <https://github.com/dmdequin/sarcopenia_and_machine_learning>.
